# Ultrahigh Performance Triboelectric Nanogenerator Enabled by Charge Transmission in Interfacial Lubrication and Potential Decentralization Design

**DOI:** 10.34133/2022/9812865

**Published:** 2022-07-05

**Authors:** Wencong He, Wenlin Liu, Shaoke Fu, Huiyuan Wu, Chuncai Shan, Zhao Wang, Yi Xi, Xue Wang, Hengyu Guo, Hong Liu, Chenguo Hu

**Affiliations:** ^1^School of Physics, State Key Laboratory of Power Transmission Equipment and System Security and New Technology, Chongqing University, Chongqing 400044, China; ^2^State Key Laboratory of Crystal Materials, Shandong University, Jinan 250100, China

## Abstract

Triboelectric nanogenerator (TENG) is a promising strategy for harvesting low frequency mechanical energy. However, the bottlenecks of limited electric output by air/dielectric breakdown and poor durability by material abrasion seriously restrict its further improvement. Herein, we propose a liquid lubrication promoted sliding mode TENG to address both issues. Liquid lubrication greatly reduces interface material abrasion, and its high breakdown strength and charge transmission effect further enhance device charge density. Besides, the potential decentralization design by the voltage balance bar effectively suppresses the dielectric breakdown. In this way, the average power density up to 87.26 W·m^−2^·Hz^−1^, energy conversion efficiency of 48%, and retention output of 90% after 500,000 operation cycles are achieved, which is the highest average power density and durability currently. Finally, a cell phone is charged to turn on by a palm-sized TENG device at 2 Hz within 25 s. This work has a significance for the commercialization of TENG-based self-powered systems.

## 1. Introduction

Triboelectric nanogenerator (TENG) is one of effective approaches by utilizing the ambient energy to power distributed sensor systems in the Internet of Things era [1–4]. Low frequency and irregular mechanical energy extensively exist in human activities and outdoor environment, and using this form of energy is a key approach for realizing the full-time self-powered sensor systems [5–8]. TENG based on coupling of triboelectrification and electrostatic induction [9, 10] has attracted plenty of research interest and shows advantages of lightweight, cost-effectiveness, and higher efficiency at low frequency region than electromagnetic generators [11–13]. Typically, a sliding mode TENG is easy to be driven by water flow and wind to produce continuous electric output, which is regarded as a promising one for practical applications [14–16]. However, the bottlenecks of electric output limited by air/dielectric breakdown in inner device [17–19] and poor durability by material abrasion during sliding motion [20, 21] seriously restrict its further improvement.

In recent years, researchers have been working on promoting electric output and durability of TENG and proposed several valuable strategies for addressing these issues. For output enhancement, efforts were dedicated to elevating the threshold of air breakdown to increase surface charge density [22], including atmosphere controlling [23–25], using thin dielectric layer with charge pumping method [26–28] and our latest reported charge space-accumulation (CSA) effect [29]. For device durability, noncontact mode [30–32], rolling friction [33, 34], soft contact with fur [35, 36], and liquid lubrication [37–40] strategies were developed. Among those strategies, liquid lubrication is the only one which reduces the material abrasion but does not sacrifice (or even increase) the electric output of the TENG device [37]. In the latest work, by introducing squalene to form interface lubrication, peak power density of the sliding mode TENG remarkably reached 3.45 W m^−2^ Hz^−1^ and retained 86% of its output after 500 k operation cycles [38]. However, its output power is still relatively low, and further boosting the output performance of TENG is highly desired.

In this work, a super durability (90% retention after 500 k cycles) and an ultrahigh peak power density (~562 W m^−2^ Hz^−1^, 163 times of the previous report [38]) are achieved based on the utilization of a novel voltage balance (VB) bar design in liquid lubrication and CSA effect. The VB bar and CSA effect in liquid lubrication suppress the dielectric breakdown and air breakdown, and liquid lubrication guarantees the super durability; all of them synergistically boost the output. The average power density reaches ~87.26 W m^−2^ Hz^−1^ through rational optimizing TENG device in silicone oil, which is comparable to the average power density of a solar cell. With this remarkable breakthrough, a cell phone is successfully charged to turn on using a palm-sized device at 120 rpm operation frequency within 25 s, indicating its high-efficiency for harvesting low frequency mechanical energy. It is a milestone for the commercialization of TENG-based self-powered systems.

## 2. Results

### 2.1. Structure Design and Output Performance of Liquid Lubrication Promoted TENG

**Figure 1:**
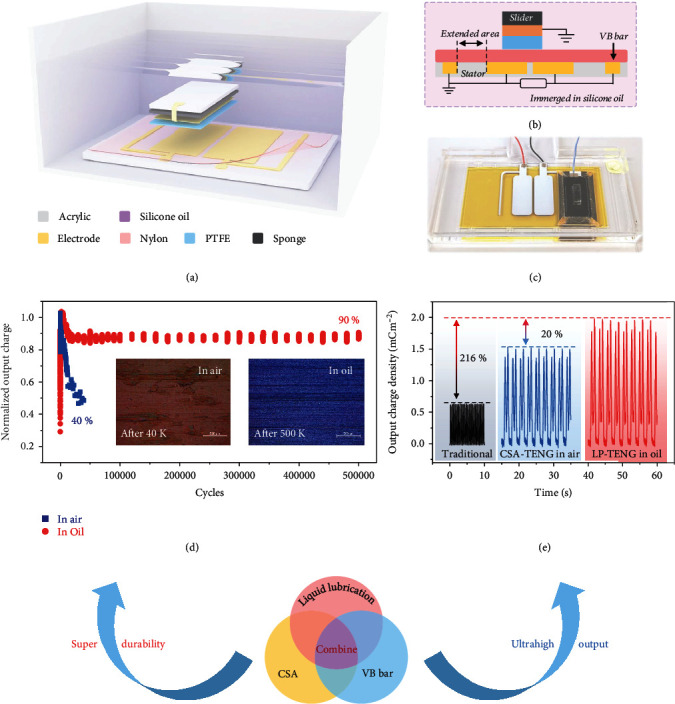
The basic device structure and performance of LP-TENG. (a) 3D schematic of the triboelectric nanogenerator immersed in silicone oil. (b) Cross-section diagram of the device component and electric connection. (c) Photograph of the basic device. (d) Electrical and mechanical durability of the device. Insets are the surface microscopic photograph of nylon layer after working in air and oil. (e) Output charge density of the device compared with traditional one.

For traditional sliding mode TENG, air breakdown effect in contact interface voids and material abrasion during the sliding motion are the main problems. In previous work, we proposed the charge space-accumulation (CSA) strategy [29] to restrain air breakdown and realize the boost in surface charge density (the fundamental mechanism is presented in Figure S1 and Note S1). Because high surface charge density induces strong electrostatic force between two tribolayers, the increase in surface friction lowers the energy conversion efficiency and destroys mechanical durability. Insulating oil is widely used in high-voltage transforming station and machinery fields. Its antidischarge protection and surface lubrication property enable it to be the best candidate for addressing the bottleneck issues in TENG field. The basic unit of our liquid lubrication promoted TENG (LP-TENG) is schematically illustrated in Figure 1(a), in which a CSA-TENG is fully immersed in silicone oil. The detailed fabrication process is described in Experimental Section. Specifically, a voltage-balance bar is designed to suppress the dielectric breakdown of tribolayer and thus further promotes the output power (Figure 1(b)), which will be discussed in the following text. Photograph of the LP-TENG is displayed in Figure 1(c). With liquid lubrication, the mechanical durability of the LP-TENG is highly improved. As shown in Figure 1(d), the electric output in silicone oil retains 90% of its initial value and keeps steady after 500 k operation cycles, while it only retains 40% after 40 k cycles in air condition. In addition, the electric output of LP-TENG is also boosted. Theoretically, output power is proportional to the square of surface charge density. As a result, the charge density of CSA-TENG in oil reaches 2.0 mC m^−2^, which increases by 20% compared with the CSA-TENG in air and by 216% compared with the traditional sliding mode TENG experimentally (Figure 1(e)).

### 2.2. Charge-Liquid Transmission

Here, besides the utilization of CSA strategy, the role of insulating oil in surface charge improvement was systematically studied. First, when operating LP-TENG device, air in the interfacial voids is squeezed out, and the voids are fully filled with the liquid medium. The pristine nonpolarized oil does not screen the surface charge, but it raises the ultimate value due to its much higher dielectric breakdown strength [40]. However, in real situations, ions, colloidal particles, or other charge carriers in the oil consume and lower the surface charge density (Figure S2). Therefore, a proper type of insulating oil is very important for surface charge improvement. In this work, by immersing two electrodes in different insulating oil and scanning in high voltage, the macroscopic behaviors of charge carriers for various liquid mediums are characterized (Figure S3). Silicone oil has the lowest conductivity and highest charge output compared with mineral oil, transformer oil, and paraffin oil adopted in LP-TENG (Figure S4). The specific output waveforms are shown in Figure S5. Other parameters of these oil are listed in Table S1 and Table S2.

**Figure 2:**
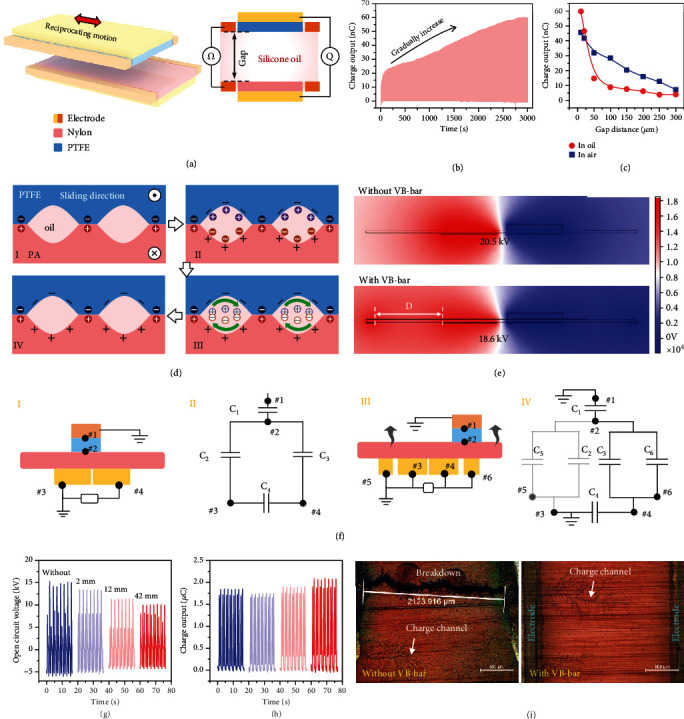
The mechanism of charge-liquid transmission effect and VB bar. (a) The experimental schematic for measuring the charge output of noncontact sliding mode TENG in insulation oil. (b) Dynamic output charge curve. The gap distance is 10 *μ*m. (c) The output charge comparison in oil and air conditions under various gap distances. (d) The schematic illustration of surface charging process in LP-TENG. The relative sliding direction is perpendicular to the cross-section. (e) Potential simulation of the basic LP-TENG with or without a voltage balance bar. (f) The structure and capacitance model of the LP-TENG with (insets I and II) or without (insets III and IV) the VB bar. (g, h) The voltage and charge output of LP-TENG with differently designed voltage balance bars. (i) The microscopic photograph of the area between two bottom electrodes after working in high impendence condition.

Second, for operating TENG in air condition, due to the existence of the inevitably interfacial voids, only the convex surface area in direct contact is charged, while the sunk surface area is not charged by triboelectrification process. Surprisingly, a sandwiched liquid layer existed between two solid tribomaterials which transmits charges (so-called charge-liquid transmission effect), so that surface charge also forms on trio-layers by the relative sliding movement between the device and liquid, though two tribolayers are not in direct contact. As schematically shown in Figure 2(a), a basic sliding mode TENG using PTFE and PA as the tribomaterials is employed for demonstrating this effect. The zero-gap position of two tribolayers is determined by detecting the resistance of two side-electrodes, and then, a precise displacement platform is used to create a certain gap distance (Figure S6). As a result, the charge output of the basic sliding mode TENG in silicone oil with 10 *μ*m gap distance increases continuously during 3000 s (Figure 2(b)). Usually, in air condition, the output charge of sliding mode TENG decreases linearly with the increase in gap distance. However, as measured in Figure 2(c), the output charge in silicone oil shows exponential decrease trends, which further proves the existence of charge-liquid interchange effect and its gap-dependent property. In this case, when operating LP-TENG, charge first forms on PTFE and PA surfaces, which is from direct contact by solid-solid electrification (Figure 2(d), I). Then, by solid-liquid electrification process, an electric double layer is created on the noncontact surface (Figure 2(d), II). Later, the flowing oil makes the opposite charges convect and recombine; thus, net charge finally forms on the noncontact surface (Figure 2(d), III and IV). In order to obtain higher output, other factors such as tribomaterial, oil viscidity, and sliding speed are presented and discussed in Figure S7 and Note S2.

### 2.3. The Mechanism of VB Bar

Proverbially, due to the capacitive feature of TENG, the highest output is always achieved under a highly matched external loads. Under optimized working conditions, with the surface charge improvement, the voltage between the bottom electrodes is huge, which causes dielectric breakdown and device failure. By introducing a voltage balance (VB) bar, with the same surface charge density, the potential difference of two electrodes is suppressed to a certain extent as simulated in Figure 2(e). It indicates that after the introduction of VB bar, the overall potential decreases significantly, and the potential difference between the bottom electrodes decreases significantly as well. The role of VB bar is to disperse the centralized potential distribution around the bottom electrodes and to lower the local electric field. The simulation parameters and more results on the position of VB bar are presented in Figure S8 and Note S3 in detail (*D* is the distance between VB bar and bottom electrodes).

From another aspect, according to the capacitance model of TENG (Equation (1)), higher surface charge density will lead to higher voltage, which will cause dielectric breakdown and limit its maximum output.

(1) $$\begin{array}{c} U=\frac{Q}{C}=\frac{\sigma S}{C}, \end{array}$$

where *U*, *Q*, and *C* are the output voltage, surface charge, and capacitance of TENG, respectively. *S* and *σ* are the surface area and charge density of tribolayer.

Therefore, for further boosting the output power, it is necessary to effectively avoid the dielectric breakdown by dispersing the output voltage while maintaining a high output charge density. For the sliding mode TENG without a VB bar, as the structure and equivalent capacitive model is shown in Figure 2(f), I and II, nodes #1 and #3 have the same potential because they are both grounded. Therefore, the potential difference between #3 and #4 (*U*_3,4_) is the same as that between #1 and #4 (*U*_1,4_). Thus, the voltage between the bottom electrodes (*U*) can be derived as follows.

(2) $$\begin{array}{c} U=U_{3,4}=U_{1,4}, \end{array}$$

(3) $$\begin{array}{c} C_{1,4}=\frac{1}{\left(1/C_{1}\right)+\left(1/C_{3}\right)}, \end{array}$$

where *C*_1_ is the equivalent capacitance between nodes #1 and #2 and *C*_3_ is the equivalent capacitance between nodes #2 and #4. *C*_1,4_ is the total equivalent capacitance between nodes #1 and #4.

For the LP-TENG with a VB bar, its structure and capacitive models are shown in Figure 2(f), III and IV. *C*_5_ and *C*_6_ are the additional capacitance from the introduction of the VB bar located on either side of the bottom electrode. Same as the sliding mode TENG without the VB bar, nodes #1 and #3 have the same potential and the voltage between the bottom electrodes (*U*′) equal to the voltage between nodes #3 and #4 (*U*′_3,4_)  as follows.

(4) $$\begin{array}{c} U^{\prime }=U\prime_{3,4}=U\prime_{1,4}, \end{array}$$

(5) $$\begin{array}{c} C_{1,4}^{\prime }=\frac{1}{\left(1/C_{1}\right)+\left(1/\left(C_{3}+C_{6}\right)\right)}, \end{array}$$

where *C*_6_ is the equivalent capacitance between nodes #2 and #6 and *C*′_1,4_ is the total equivalent capacitance between nodes #1 and #4 for LP-TENG.

Comparing *C*_1,4_ in Equation (3) with *C*′_1,4_ in Equation (5), the capacitance value between nodes #1 and #4 increases by introducing the VB bar. According to Equation (1), the output voltage is inversely proportional to the capacitance *C*. Therefore, as the total capacitance between #1 and #4 increases due to the *C*_6_ in Equation (5), the output voltage becomes smaller. Besides, as *D* increases, the charge space accumulation area between #4 and #6 increases accordingly. When the slider reaches #6 from #4, the surface charge also increases due to the larger charge space accumulation area. Hence, at position #6, it induces more charge on VB bar. Therefore, *C*_6_ increases with the increase in *D*, and *C*′_1,4_ also increases with the increase of *C*_6_. In this way, the VB bar does effectively disperse the voltage between the bottom electrodes, which greatly inhibits dielectric breakdown.

Experimentally, the open-circuit voltage of LP-TENG with different *D* values of the VB bar was measured, which matches well with the simulated results (Figure 2(g)). The actual devices for the experiment are illustrated in Figure S9. With the increase in *D*, open-circuit voltage of LP-TENG decreases (from 15 kV to 10 kV), while the short-circuit charge transfer does not decrease and even exhibits a small incremental behavior (Figure 2(h)). Figure 2(i) demonstrates the microscopic images of the area between two bottom electrodes when operating LP-TENG under high external impedance (100 G ohm) with (right one) and without (left one) VB bar, from which, we can see that the dielectric breakdown occurs and a direct conductive route forms between two electrodes without employing VB bar. While the dielectric breakdown is restrained, it only forms some charge diffusion channels by utilizing VB bar. Therefore, benefiting from the surface charge improvement and suppressed dielectric breakdown, the boosting output power is guaranteed. Finally, we measured the electric output performance of LP-TENG with silicone oil lubrication under various external load resistance as shown in Figure S10, where the maximized peak power density reaches 4.43 W m^−2^ Hz^−1^ at 100 M*Ω* external load, which is rather high for a single liquid lubricating S-TENG unit.

### 2.4. Super Durability and Ultrahigh Output Performance

**Figure 3:**
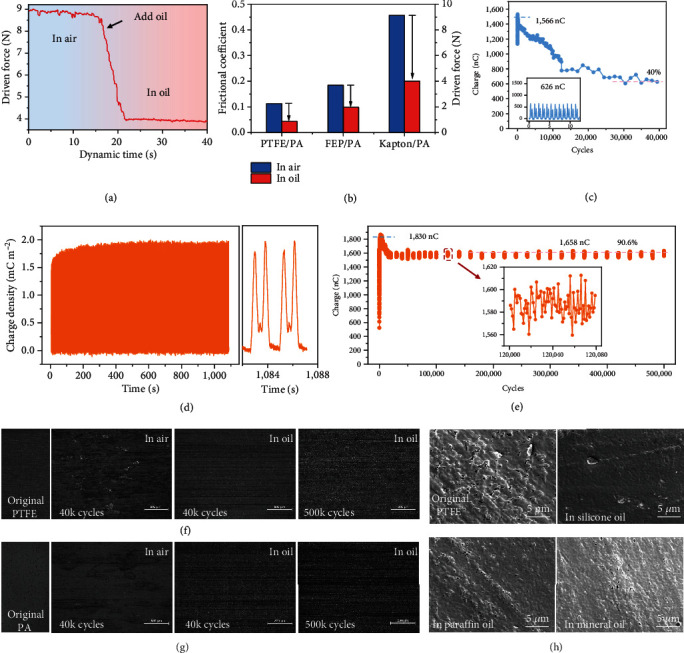
Lubricity and durability of LP-TENG. (a) The dynamic driving force of a sliding mode TENG after adding silicone oil. (b) The driving force and corresponding equivalent frictional coefficient of the sliding mode TENG with different tribomaterials. (c) The durability of S-TENG in air within 40 k cycles (0.1 m s^−1^). Inset is the charge output of S-TENG after all cycles. (d) The dynamic charge density of LP-TENG in silicone oil (0.1 m s^−1^). The enlarged image on the right shows the specific waveform. (e) The durability of LP-TENG within 500 k cycles at 0.1 m s^−1^. The illustration is a larger view of the red dotted box. The microscopic images of (f) PTFE layer and (g) PA layer during long-term operation cycles in air and oil conditions (scale bar: 500 *μ*m). (h) The SEM images of PTFE treated with different liquid.

On the other hand, the lubricity of insulating oil lowers the friction force between solid-solid interface and thus decreases the idle heat-work from overcoming friction and increases the energy conversion efficiency of TENG device. In this work, by fixing the vertical pressure as 20 N, the dynamic force for driving the sliding part is recorded as shown in Figure 3(a). The schematic of testing setup is depicted in Figure S11. For Kapton/PA tribomaterials, after adding silicone oil, the driving force dramatically decreases from 9.0 N to 4.0 N. In this way, the driving force of different tribomaterials (PTFE/PA, FEP/PA, and Kapton/PA) and their equivalent frictional coefficient can be derived (Figure 3(b)). The results show that the frictional coefficient of all the materials with liquid lubrication is lower than that in air. Besides, the friction coefficient of PTFE decreases as the load increases within a certain load range, which indicates that a modest increase in pressure can improve contact without dramatically increasing wear. Typically, due to certain lubricating property of PTFE, the frictional coefficient of PTFE/PA interface employed in LP-TENG is 0.11 (the smallest) in air and decreases to 0.04 in oil condition, which is the foundation for realizing a higher energy conversion efficiency. The detailed driving force and frictional coefficient of different materials are shown in Figure S12. The vertical pressure acting on the slider can be adjusted by the experimental platform (Figure S13), and 8.7 N is applied on LP-TENG in this work.

To achieve super durability without sacrificing its output performance, first, the durability of the S-TENG in the air is characterized as shown in Figure 3(c). Obviously, its output charge decreases sharply from 1566 to 626 nC after 40 k operation cycles, indicating only 40% retention. The initial output waveform of this test is shown in Figure S14 and S15. In the air, the frictional coefficient between the tribolayers is large, and the wear at the interface is serious. The surface microstructure of the original PTFE displays shallowly aligned fine lines (Figure S16(a, b)). After 40 k cycles in the air, there are many concave scratches and wear debris distributed on the surface of PTFE including debris from tribolayers and formed anomalous convex structures, which leads to a further reduction in the effective working area (Figure S16(c, d)). As a large number of air voids exist in the contact interface due to the surface feature of the two tribolayers, the maximum output in the air is limited by air-breakdown in voids and by decrease in working area due to wear debris. As a result, the S-TENG has poor durability in the air.

However, the air voids on the interface are filled when the lubrication liquid is introduced. Combined with the liquid triboelectrification and charge transmission mechanism described above, the maximum output charge density of 1.96 mC m^−2^ for the sliding mode LP-TENG is achieved, as shown in Figure 3(d). Its output voltage and current are plotted in Figure S17. And then, the durability of LP-TENG is characterized as shown in Figure S18 and Figure 3(e). The surface structure of the tribomaterials is characterized after each independent durability test. It is found that the wear marks on the surface of the tribomaterials gradually increase from 1 k to 20 k cycles, but the surface wear tends to be constant after that as shown in Figure S19, and its output barely decreases further. However, during the sliding motion in the air, local heat and surface abrasion cause material transfer into each other, which screens the surface charge and breaks device electric output. Nevertheless, insulating oil conducts heat efficiently and reduces material abrasion and cleans up surface residue simultaneously. As microscopic images demonstrate in Figures 3(f) and 3(g), after 40 k cycles, the surfaces of PTFE and PA are both destroyed and attached with the other fragments in the air, while, after 40 k and even 500 k cycles in oil, there only exist some scratches on the tribosurface, which ensures the long-term stability of LP-TENG. The output charge of LP-TENG maintains 90% of the maximum output after 500 k cycles, and its output is still greater than the maximum that can be achieved in the air. The output charge and current at the initiation, maximum, and end of this test are shown in Figure S20. Notably, 10% decay almost happens in the initial 20 k cycles, and then, the LP-TENG keeps 100% stable as shown in Figure 3(e) and the full graph and detail as shown in Figure S21. However, in the previous report, because a small amount of lubrication liquid used in operation process was not able to clean up the surface debris, the output showed continuously degenerative trends [39]. In addition, by comparing the scanning electron microscopy (SEM) images of nylon and PTFE treated with different lubrication liquids, we found that the lubrication liquids have a certain effect on the removal of the protrusions on the surface of the materials, especially the mineral oil as shown in Figure S22. It indicates that the lubrication liquid (especially the silicone oil) can repair the holes on the PTFE surface, making it smoother and improving the effective contact area (Figure 3(h)). Therefore, based on the compatibility of tribolayer material with lubrication liquid and combined with reasonable structural design, the LP-TENG with ultrahigh output and super durability is obtained.

### 2.5. Application Demonstration of Rotational LP-TENG

**Figure 4:**
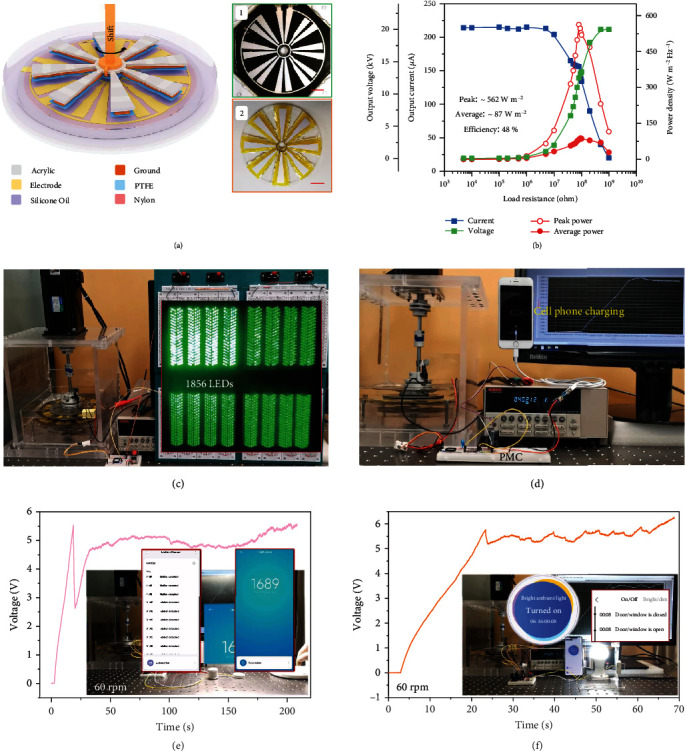
The output capability of a rotational LP-TENG. (a) 3D schematic of a rotational LP-TENG. Adjacent bottom electrodes are equivalent to the voltage-balance bar in basic LP-TENG. Insets 1 and 2 show the photographs of the stator and rotator components (scale bar: 2.5 cm). (b) The matched impendence and maximized output measurement of LP-TENG at the speed of 60 rpm. (c) Instantaneously lighting up thousands of LEDs under 60 rpm. (d) Continuously charging a cell phone after power management at 120 rpm. (e) The motion sensor and light sensor powered by LP-TENG sustainably and received the signals by wireless transmission. (f) A wireless switch sensor can be powered by LP-TENG with 3.3 mF capacitor at 60 rpm and controlling the bulb intelligently by accessing the local area network (LAN) via wireless.

With the improvement in electric output and device durability, we further prepared a rotational LP-TENG (diameter: 15 cm) for demonstrating its capability in low frequency mechanical energy harvesting. As shown in Figure 4(a), due to the centrosymmetry of the rotational device, adjacent bottom electrodes play the role of the VB bar. The photographs of the stator, rotator, and entire device are shown in inset 1, inset 2, and Figure S23, respectively. The output charge at the rotation speed from 15 to 120 rpm was measured, and the maximum charge transfer of 6932 nC (2.0 mC m^−2^) and open-circuit voltage over 20 kV are achieved at 60 rpm (Figure S24), which is rather high for a rotary mode TENG. Figure 4(b) shows the output power density, current, and voltage under various external load resistance ranging from 5 k*Ω* to 1 G*Ω*. The optimized peak power density and average power density reach 562.36 W m^−2^ Hz^−1^ and 87.26 W m^−2^ Hz^−1^ with the matching impedance of ~80 M*Ω*, respectively. The maximum peak power density is 163 times enhancement compared with previous reports [38]. And the current crest factor is about 1.43. Considering the equivalent frictional coefficient as 0.04 and using the average power for estimating, the energy conversion efficiency up to 48.61% is obtained according to the following equation (Note S4 for details).

(6) $$\begin{array}{c} \eta =\frac{W_{e}}{E_{f}+W_{e}+E_{k}}=\frac{P_{av}\cdot t}{\left(4/3\right)\pi \mu F_{N}\left(\left({R_{2}}^{3}-{R_{1}}^{3}\right)/\left({R_{2}}^{2}-{R_{1}}^{2}\right)\right)+P_{av}\cdot t+\left(1/4\right)m\left({R_{1}}^{2}+{R_{2}}^{2}\right)\omega^{2}}, \end{array}$$

where *W*_*e*_, *E*_*f*_, *P*_*av*_, *t*, *μ*, *F*_*N*_, *m*, *ω*, and *R*_1_ and *R*_2_ are the output electricity in one rotation, the energy used to fight against the friction in one rotation, the average output power, time, the dynamic friction factor, the vertical pressure, the mass of rotor, the angular velocity, and the inner and outer radius of the rotor, respectively.

From the recent important works on improving performance of sliding mode TENG summarized in Table S3, we can see the outstanding achievement of our proposed LP-TENG. For practical applications, the output of rotational LP-TENG is first used to charge different capacitors with rectifier circuit at rotating speed of 60 rpm as shown in Figure S25. The capacitor of 1 *μ*F and 10 *μ*F is charged to 200 V within 7.5 s and 36 s, respectively. The large voltage steps indicate the ultrahigh output capacity of the rotational LP-TENG again. And then, bigger capacitors are charged to further demonstrate its capability of energy output. The 1 mF and 3.3 mF capacitors can be charged to 7 V and 5.7 V within 104 s and 310 s, respectively, illustrating the ultrahigh charging efficiency. Second, the rotational LP-TENG directly lights up over 1850 LEDs at 60 rpm (Figure 4(c) and Movie S1) and powering two hygrothermometers for 3 min at 45 rpm (Movie S2 and Figure S26). Third, combined with a matched power management circuit (PMC), a cell phone is charged continuously without any mechanical transmission components under the low operation frequency of 120 rpm (Figure 4(d) and Movie S3). The charging curve and power management circuit are depicted in Figure S27 and Figure S28. As a reference, the classic work of using the rotary mode TENG for charging cell phone was realized at a high frequency of 3000 rpm [41]. Fourth, the LP-TENG sustainably powers the motion sensor and light sensor with wireless transmission in parallel by 3.3 mF capacitor with PMC at 60 rpm (Figure 4(e)). When a person moves and operates the lamp in front of the sensors, the sensors work and transmit signals wirelessly as shown in Movie S4. Last, a wireless switch and a light sensor are also powered by LP-TENG with 3.3 mF capacitor at 60 rpm (Figure 4(f) and Movie S5), by which we can control the smart bulb by turning on and off the switch and receive the switch signal remotely. These practical applications indicate that our work provides an effective strategy for achieving high-output and high durability TENG towards practical applications.

## 3. Conclusion

We have demonstrated a feasible strategy for improving both the electric output and durability of the sliding mode TENG. By rationally designing a voltage balance bar, utilizing charge space accumulation mechanism, high dielectric strength, charge-liquid transmission effect, and lubricity of insulating oil, our LP-TENG achieves an average power density up to 87.26 W·m^−2^·Hz^−1^, the energy conversion efficiency of 48%, and output retention of 90% after 500 k operation cycles. The average power density is the largest one at the present stage in TENG field, which is comparable to that of a solar cell. By developing high-precision device processing technique, the energy conversion efficiency and durability could be improved further. Our strategy can be employed in other mode TENG designs to solve their inner dielectric breakdown and stability issues. With these capabilities, TENG will be a viable technology for harvesting low-frequency mechanical energy and for building the full-time self-powered systems.

## 4. Experimental Section

### 4.1. Fabrication of the LP-TENG

For the stator, (i) an acrylic plate with the size of 155 × 77 × 3 mm (length × width × thickness, the same for the following description) was prepared by laser cutter as the substrate. (ii) After being attached with a 15 *μ*m aluminum (Al) film, two chamfered bottom electrodes (21 × 50 mm, rounded corner: 4 mm) and their voltage balance bar (2 × 50 mm) were patterned by knife cutting. The gap distance of two bottom electrodes was fixed as 2 mm. For comparison, the voltage balance bar was placed 2 mm, 12 mm, and 42 mm from its adjacent bottom electrode. The entire conductive layer was aligned in the center of the substrate. (iii) Finally, a 25 *μ*m nylon film was adhered and fully covered the substrate using epoxy resin to seal its edge. For the slider, (i) before covering a buffer sponge layer (stiffness: 30 Psi; size: 39 × 66 × 1.5 mm), a rectangular acrylic sheet (39 × 66 × 3 mm) was fabricated as the substrate by laser cutter. (ii) Then, a chamfered Al electrode (21 × 50 mm, rounded corner: 4 mm) was stuck on the sponge surface, and a tail was curved out for connecting with ground. (iii) Finally, the whole component was attached and encapsulated with a 33 *μ*m PTFE film (58 × 89 mm).

### 4.2. Fabrication of the Rotary LP-TENG

For the stator, (i) a 3 mm (thickness) square acrylic plate with the width of 18 cm was prepared as the substrate, and a hole with diameter of 12 mm was cut by laser cutter in the center of the substrate for placing the bearing. (ii) Then, the substrate was carved with tiny radial electrode traces by laser cutter with an inner diameter of 18 mm and outer diameter of 15 cm. Next, a 20 *μ*m Al film was pasted on the substrate and carved according to those marks, leaving the electrode, inner gap, and blank area with the central angle of 10°, 5°, and 20°, respectively. (iii) For optimizing the contact, a 1.5 mm chamfered square sponge with length of 17 cm (rounded corner: 4 cm) was sandwiched between Al electrode and the substrate. Then, a nylon film of the same size (25 *μ*m in thickness) was adhered to overall surface. At last, we sealed the perimeter with epoxy resin. For the rotator, (i) a 3 mm acrylic disc with the diameter of 16 cm was prepared as the backboard of the rotator. (ii) The acrylic plate was cut into 8 sectors with size of 2.25 cm, 15 cm, and 20° for its inner diameter, outer diameter, and center angle, respectively, as the electrode substrates. (iii) Then, the electrode profiles were carved on these substrates with size of 3 cm, 14.4 cm, and 10°, for its inner diameter, outer diameter, and center angle, respectively. Then, 15 *μ*m Al film was stuck on the electrode profile. The total area of those electrodes is about 34.62 cm^2^. (iv) For the tribolayer, 33 *μ*m PTFE films were applied to the surface of sectors, and their perimeters ware sealed with epoxy resin.

### 4.3. Electric Measurement and Characterization

All the devices were fixed and tested on an optical platform (ZPT-G-M-15-10). The slider of LP-TENG was driven by a linear motor (LinMot PS01-37 × 120-C). A commercial programmable stepper motor (86HSE12N) was used to drive the rotary LP-TENG. The current, transferred charge, and voltage of capacitors were measured by an electrometer (Keithley 6514), and the load voltage was measured by a high-speed electrostatic voltmeter (Trek Model 370) with series resistance voltage division method. The surface microstructure photos of tribolayer were taken by an upright metallurgical microscope (Leica DM2700M). The room temperature (15-28°C) and humidity (45%-85% RH) were measured by a commercial thermohygrometer (TH20R).

## Data Availability

The data that support the findings of this study are available from the corresponding authors upon reasonable request.

## Supplementary Materials

Supplementary Materials Figure S1: fundamental mechanism of charge space accumulation (CSA) strategy. Figure S2: the conductive carriers in oil that affect surface charge of LP-TENG. Figure S3: schematic of the system for testing the I-V curve of the insulating oil. Figure S4: I-V curve of the insulating oil and charge output of LP-TENG under different lubrication liquid. Figure S5: the output performance of LP-TENG with different lubrication liquid. Figure S6: verifying the charge-liquid transmission effect. Figure S7: other factors that affect the output of LP-TENG. Figure S8: simulation of the effect of VB bars. Figure S9: the device design parameters of VB bar for experimental investigation. Figure S10: matching impendence measurement of LP-TENG at speed of 0.1 m s^−1^. Figure S11: driving force and equivalent frictional coefficient test of LP-TENG. Figure S12: tribological characterization of different materials. Figure S13: adjustable load pressure. Figure S14: the output waveforms of S-TENG in air at different stage in the cycles. Figure S15: the output performance of S-TENG in air. Figure S16: characterization of the surface wear for PTFE. Figure S17: the output performance of LP-TENG. Figure S18: the long-term durability of LP-TENG. Figure S19: the surface microscopic images of PTFE after different operation cycles. Figure S20: the output waveforms of LP-TENG at different stage in the 500,000 cycles. Figure S21: the detail waveform of 500,000 cycles durability test. Figure S22: the SEM images of PTFE and nylon treaded by different liquid. Figure S23: photograph of rotational LP-TENG. Figure S24: the output performance of rotary LP-TENG. Figure S25: the voltage curves of charging different capacitors. Figure S26: powering two commercial hygrothermometers. Figure S27: the charging curve of a 3.3 mF capacitor during the cell phone charging process. Figure S28: the circuit diagram and photo of power management circuit. Table S1: the parameters of lubrication liquids. Table S2: parameters of silicone oil with different viscosity. Table S3: output performance comparison with the reported S-TENG. Note S1: the mechanism of charge space accumulation (CSA) strategy. Note S2: effect of materials, oil viscosity, and sliding speed on the output of LP-TENG. Note S3: simulation of the effect of VB bar. Note S4: evaluating the energy conversion efficiency and crest factor. Other supporting materials for this work includes the following: Movie S1: lighting up 1856 LEDs by LP-TENG (.mp4). Movie S2: powering two hygrothermometers by LP-TENG (.mp4). Movie S3: charging cell phone by LP-TENG after PMC (.mp4). Movie S4: powering wireless motion sensor and light sensor.
