# Supplementary material for: Ultrahigh Performance Triboelectric Nanogenerator Enabled by Charge Transmission in Interfacial Lubrication and Potential Decentralization Design
Source: Research (Wash D C). 2022 Jul 5;2022:9812865. doi: 10.34133/2022/9812865 (PMC9285635; doi:10.34133/2022/9812865)
Supplement: Supplementary Materials — Figure S1: fundamental mechanism of charge space accumulation (CSA) strategy. Figure S2: the conductive carriers in oil that affect surface charge of LP-TENG. Figure S3: schematic of the system for testing the I-V curve of the insulating oil. Figure S4: I-V curve of the insulating oil and charge output of LP-TENG under different lubrication liquid. Figure S5: the output performance of LP-TENG with different lubrication liquid. Figure S6: verifying the charge-liquid transmission effect. Figure S7: other factors that affect the output of LP-TENG. Figure S8: simulation of the effect of VB bars. Figure S9: the device design parameters of VB bar for experimental investigation. Figure S10: matching impendence measurement of LP-TENG at speed of 0.1 m s−1. Figure S11: driving force and equivalent frictional coefficient test of LP-TENG. Figure S12: tribological characterization of different materials. Figure S13: adjustable load pressure. Figure S14: the output waveforms of S-TENG in air at different stage in the cycles. Figure S15: the output performance of S-TENG in air. Figure S16: characterization of the surface wear for PTFE. Figure S17: the output performance of LP-TENG. Figure S18: the long-term durability of LP-TENG. Figure S19: the surface microscopic images of PTFE after different operation cycles. Figure S20: the output waveforms of LP-TENG at different stage in the 500,000 cycles. Figure S21: the detail waveform of 500,000 cycles durability test. Figure S22: the SEM images of PTFE and nylon treaded by different liquid. Figure S23: photograph of rotational LP-TENG. Figure S24: the output performance of rotary LP-TENG. Figure S25: the voltage curves of charging different capacitors. Figure S26: powering two commercial hygrothermometers. Figure S27: the charging curve of a 3.3 mF capacitor during the cell phone charging process. Figure S28: the circuit diagram and photo of power management circuit. Table S1: the parameters of lubrication liquids. Table S2: parameter [file 9812865.f1.zip › Hu_Supplementary Materials.pdf]

Supplementary Materials

Ultrahigh Performance Triboelectric Nanogenerator Enabled by Charge Transmission in Interfacial Lubrication and Potential Decentralization Design

Wencong He[1], Wenlin Liu[1], Shaoke Fu[1], Huiyuan Wu[1], Chuncai Shan[1], Zhao Wang[1], Yi Xi[1], Xue Wang[1], Hengyu Guo[1,]*, Hong Liu[2,]* and Chenguo Hu[1,]*

[1] *School of Physics, State Key Laboratory of Power Transmission Equipment and System Security and New Technology, Chongqing University, Chongqing, 400044, P. R. China.*

[2] *State Key Laboratory of Crystal Materials, Shandong University, Jinan 250100, P.R. China.*

* Correspondence should be addressed to Hengyu Guo; physghy@cqu.edu.cn, and Hong Liu; hongliu@sdu.edu.cn, and Chenguo Hu; hucg@cqu.edu.cn.

This PDF including:

Figure S1. Fundamental mechanism of charge space accumulation (CSA) strategy.
Figure S2. The conductive carriers in oil that affect surface charge of LP-TENG.
Figure S3. Schematic of the system for testing the I-V curve of the insulating oil.
Figure S4. I-V curve of the insulating oil and charge output of LP-TENG under different lubrication liquid.
Figure S5. The output performance of LP-TENG with different lubrication liquid.
Figure S6. Verifying the charge-liquid transmission effect.
Figure S7. Other factors that affect the output of LP-TENG.
Figure S8. Simulation of the effect of VB bars.
Figure S9. The device design parameters of VB bar for experimental investigation.
Figure S10. Matching impendence measurement of LP-TENG at speed of 0.1 m s^{-1}.
Figure S11. Driving force and equivalent frictional coefficient test of LP-TENG.
Figure S12. Tribological characterization of different materials.
Figure S13. Adjustable load pressure.
Figure S14. The output waveforms of S-TENG in air at different stage in the cycles.
Figure S15. The output performance of S-TENG in air.
Figure S16. Characterization of the surface wear for PTFE.
Figure S17. The output performance of LP-TENG.
Figure S18. The long-term durability of LP-TENG.
Figure S19. The surface microscopic images of PTFE after different operation cycles.
Figure S20. The output waveforms of LP-TENG at different stage in the 500,000 cycles.
Figure S21. The detail waveform of 500,000 cycles durability test.
Figure S22. The SEM images of PTFE and nylon treaded by different liquid.
Figure S23. Photograph of rotational LP-TENG.
Figure S24. The output performance of rotary LP-TENG.
Figure S25. The voltage curves of charging different capacitors.
Figure S26. Powering two commercial hygro-thermometers.
Figure S27. The charging curve of a 3.3 mF capacitor during the cell phone charging process.
Figure S28. The circuit diagram and photo of power management circuit.

Table S1. The parameters of lubrication liquids.
Table S2. Parameters of silicone oil with different viscosity.
Table S3. Output performance comparison with the reported S-TENG.
Note S1. The mechanism of charge space accumulation (CSA) strategy.
Note S2. Effect of materials, oil viscosity and sliding speed on the output of LP-TENG.
Note S3. Simulation the effect of VB bar.
Note S4. Evaluating the energy conversion efficiency and crest factor.

Other Supporting Materials for this work includes the following:

Movie S1. Lighting up 1856 LEDs by LP-TENG (.mp4).
Movie S2. Powering two hygrothermometers by LP-TENG (.mp4).
Movie S3. Charging cell phone by LP-TENG after PMC (.mp4).
Movie S4. Powering wireless motion sensor and light sensor (.mp4).
Movie S5. Powering wireless switch sensor (.mp4).

Supplementary Figures

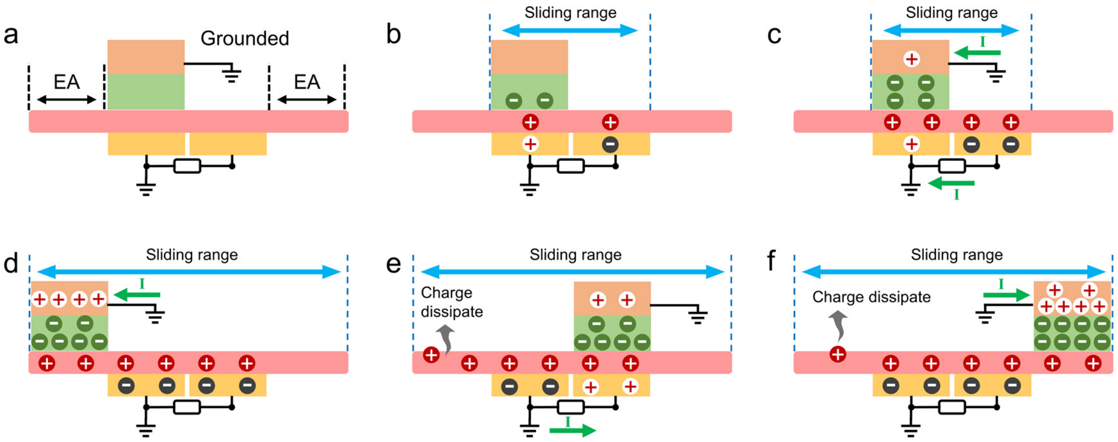

Figure S1. Fundamental mechanism of charge space accumulation (CSA) strategy. (a) The basic structure of CSA-TENG. Charge distribution (b) with and (c) without the grounded electrode when the sliding range is limited on the surface overlapped bottom electrodes. (d-f) Charge accumulation process when the sliding range is extended.

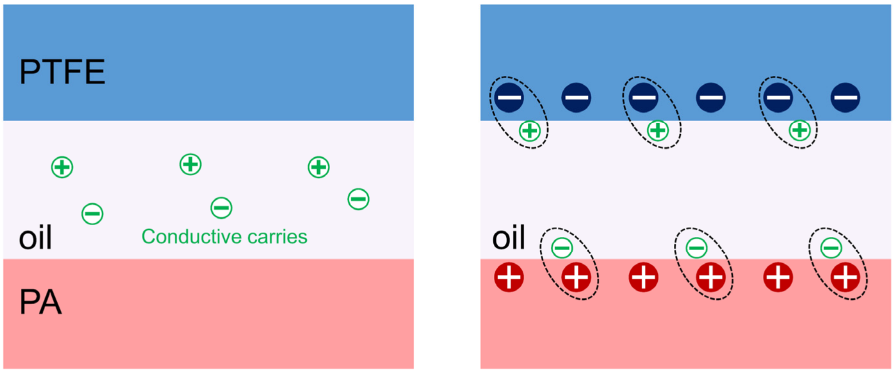

Figure S2. The conductive carriers in oil that affect surface charge of LP-TENG.

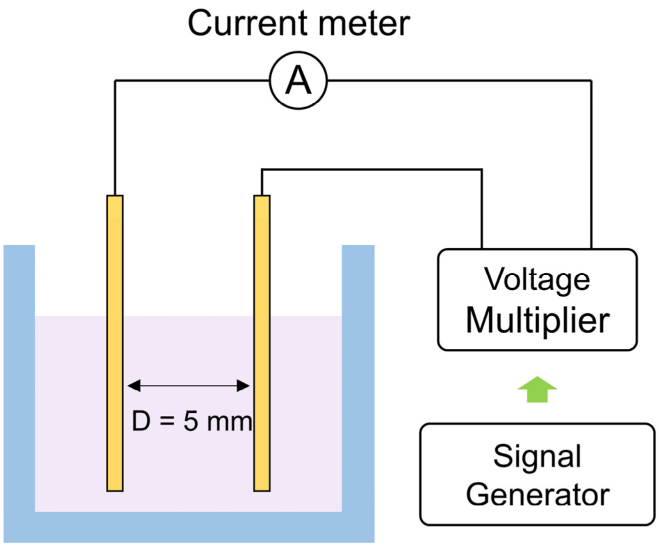

Figure S3. Schematic of the system for testing the I-V curve of the insulating oil.

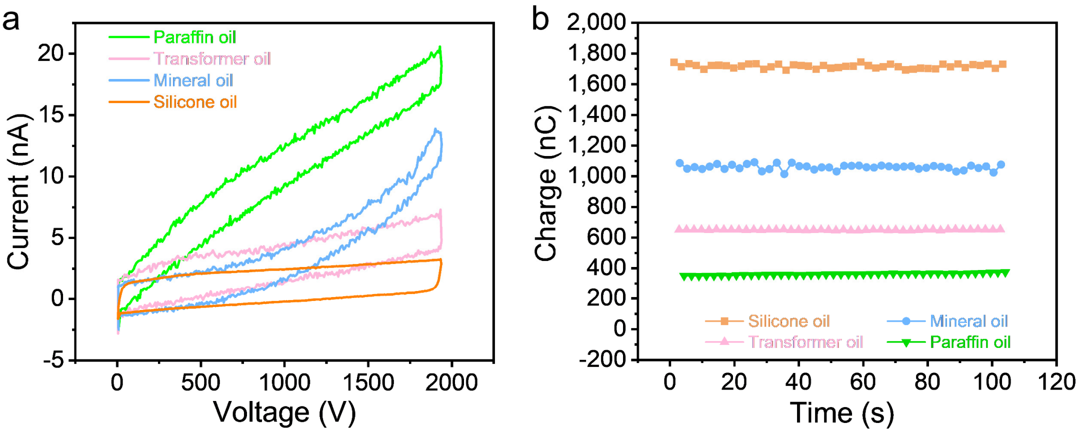

Figure S4. I-V curve of the insulating oil and charge output of LP-TENG under different lubrication liquid. (a) Cyclic voltammetry curve of different insulating oil and (b) the charge output performance of LP-TENG under different lubrication liquid.

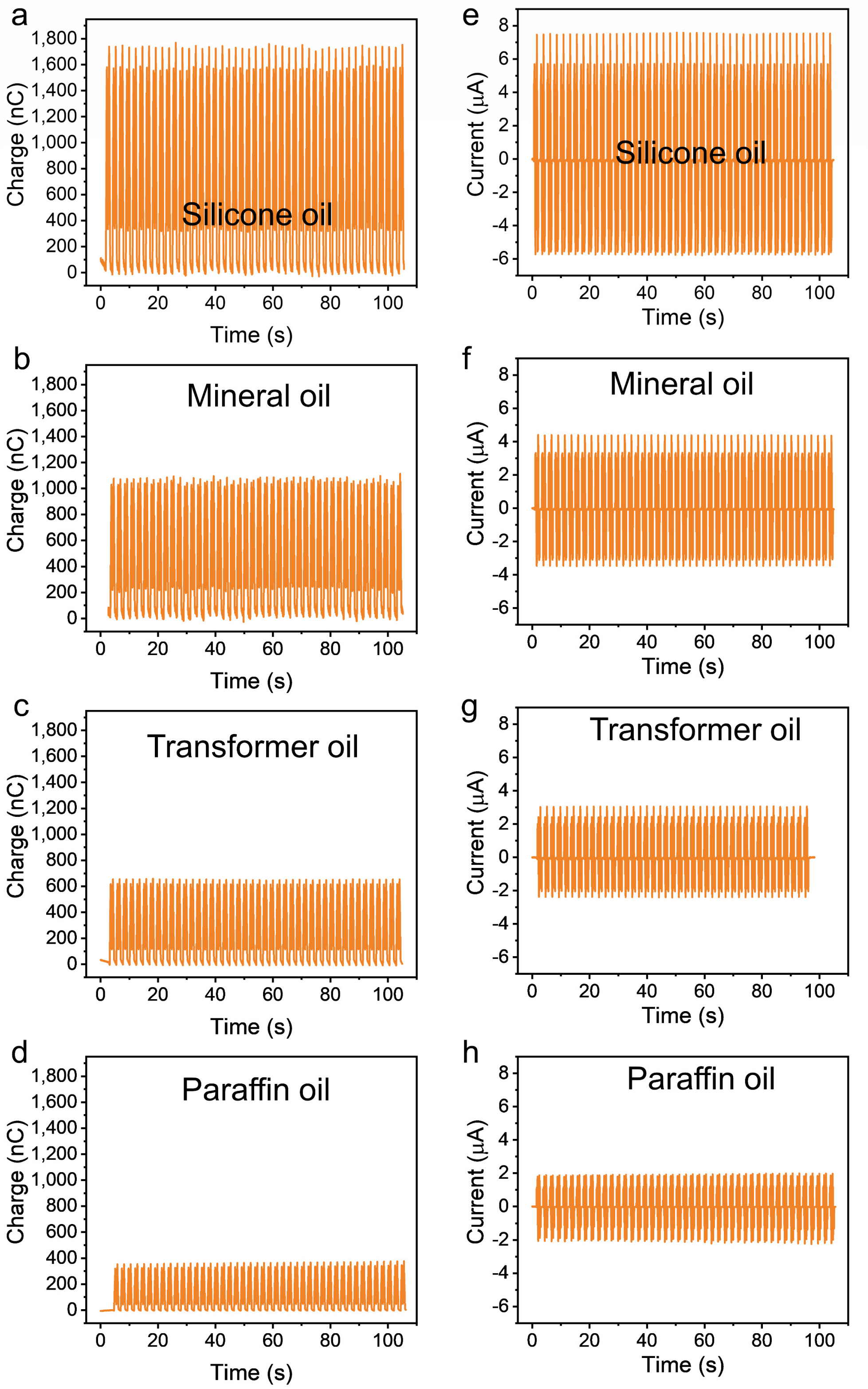

Figure S5. The output performance of LP-TENG with different lubrication liquid. The waveforms of (a-d) output charge and (e-h) short-circuit current of LP-TENG with silicone oil, mineral oil, transformer oil and paraffin oil, respectively.

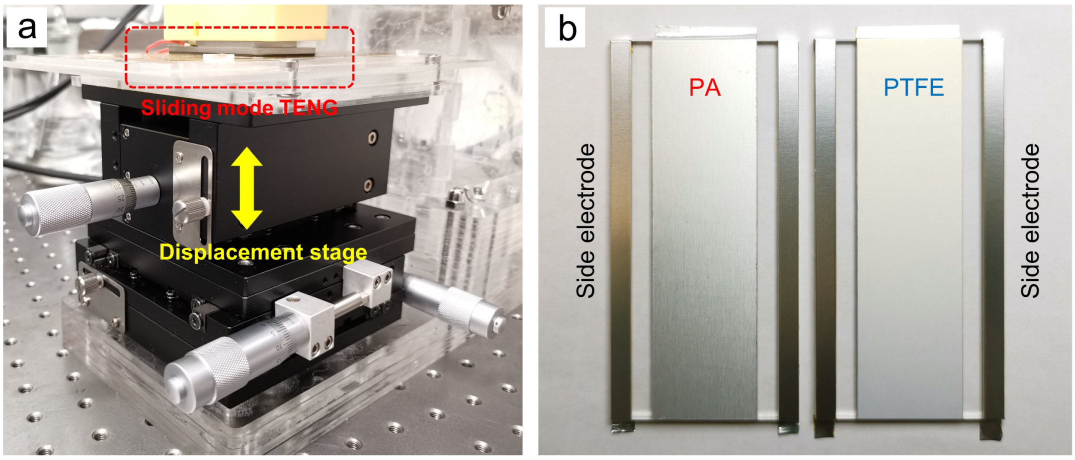

Figure S6. Verifying the charge-liquid transmission effect. (a) Experimental setup, including a precise displacement stage for creating a certain gap distance. (b) Photograph of a basic sliding mode TENG with side electrodes for confirming zero-gap position.

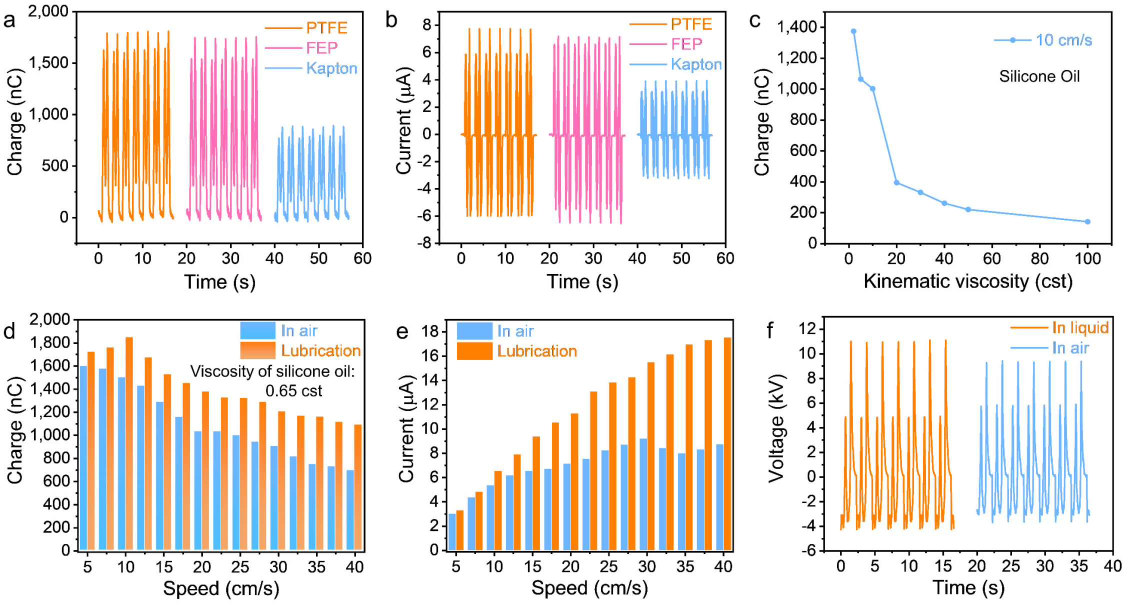

Figure S7. Other factors that affect the output of LP-TENG. (a,b) Charge and current output of different sliding materials. (c) Charge output with various silicone oil viscosity at a fixed sliding speed of 10 cm/s. (d,e) Charge and current output at various sliding speeds with the viscosity of silicone oil as 0.65 cst. (f) The voltages of LP-TENG in liquid or air.

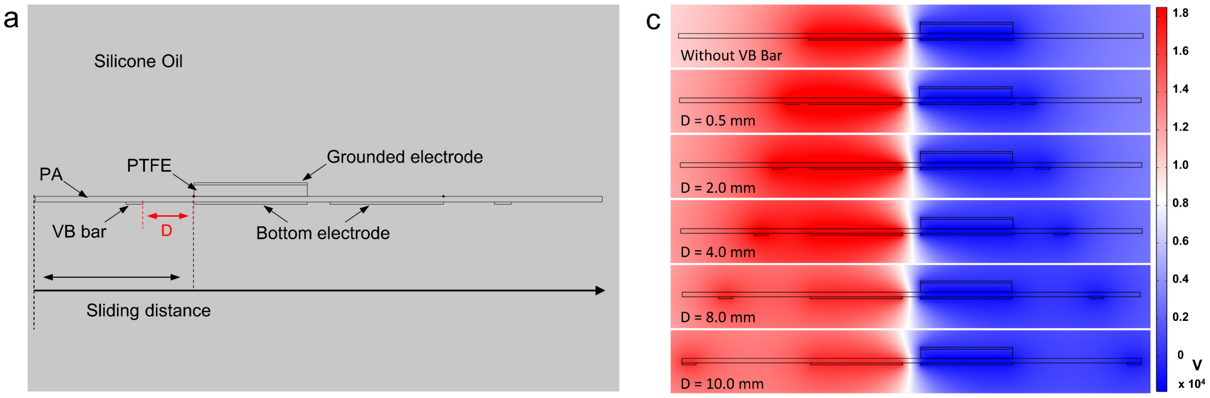

b

Parameters for simulation	
Silicone oil	Permittivity: 2.8
PTFE	Thickness: 1 mm; width: 10 mm<br>Permittivity: 4<br>Surface charge density: -1e^{-4} C/m^2
PA	Thickness: 0.5 mm; width: 50 mm<br>Permittivity: 3<br>Surface charge density(over bottom electrodes): 5e^{-6} C/m^2<br>Surface charge density(other area): 0 C/m^2
Electrode	Thickness: 0.2 mm, width: 10 mm (bottom and ground)<br>Thickness: 0.2 mm, width: 0.5 mm (VB bar)<br>Permittivity: 99999

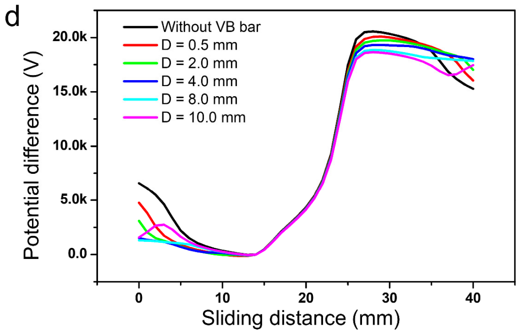

Figure S8. Simulation of the effect of VB bars. (a) Simulation mode. (b) The related simulation parameters for each component. (c) Potential distribution of LP-TENG without and with VB bar in various D values at the sliding distance of 26 mm. (d) Simulated voltage output of LP-TENG without and with VB bar in various D values in a half sliding cycle.

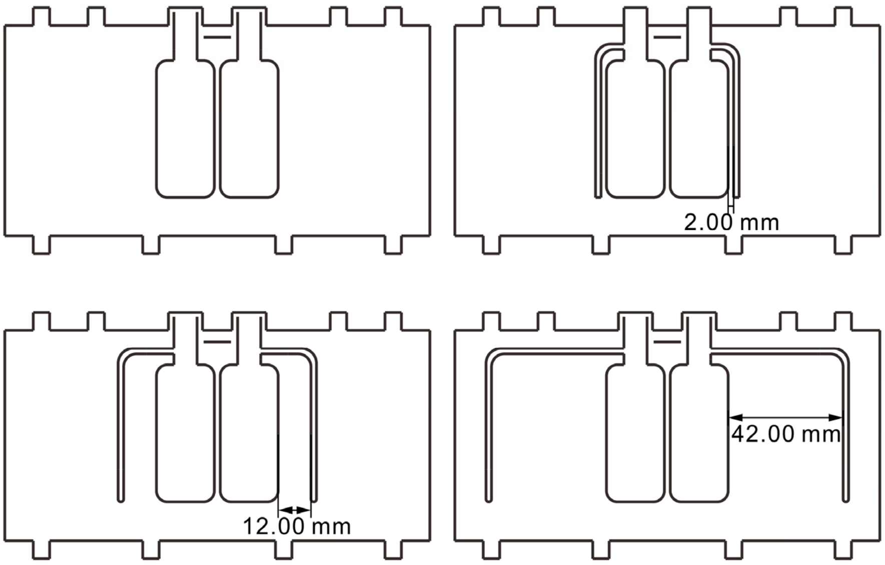

Figure S9. The device design parameters of VB bar for experimental investigation.

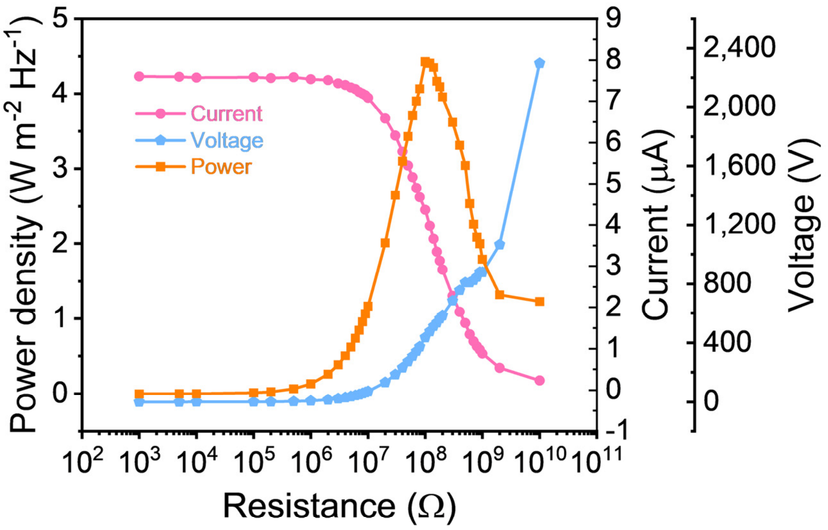

Figure S10. Matching impendence measurement of LP-TENG at speed of 0.1 m s^{-1}.

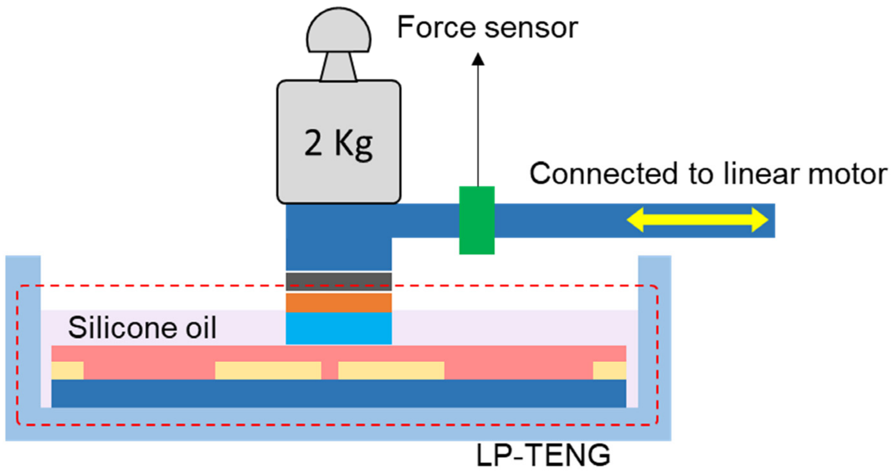

Figure S11. Driving force and equivalent frictional coefficient test of LP-TENG.

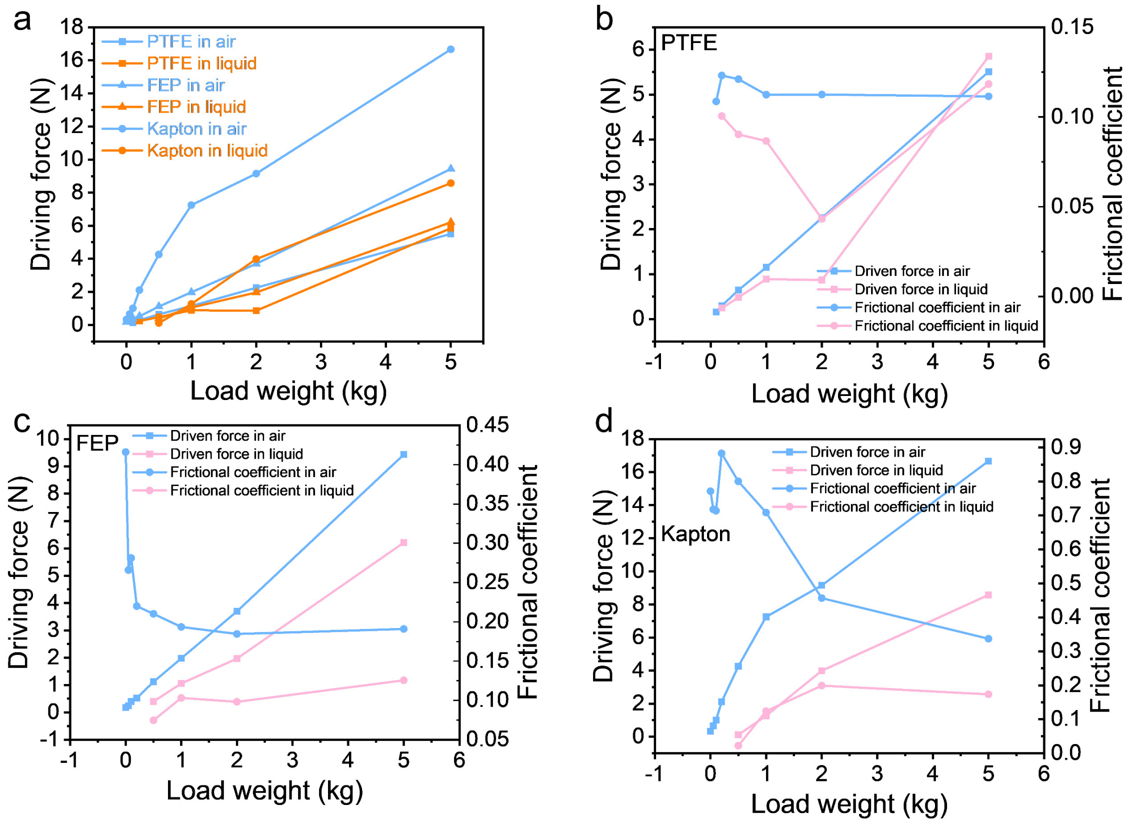

Figure S12. Tribological characterization of different materials. (a) The driving force of different materials with different load in liquid (silicone oil) and air. The driving force and frictional coefficient of (b) PTFE, (c) FEP and (d) Kapton in liquid and air.

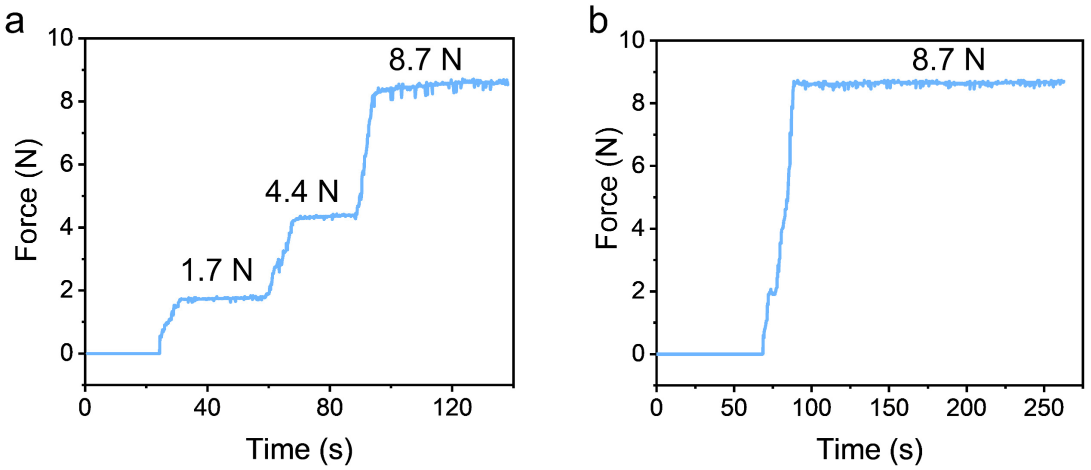

Figure S13. Adjustable load pressure. (a) Different load pressures (1.7 N, 4.4 N, 8.7 N) are applied by adjusting the platform. (b) The load we used in our experiment.

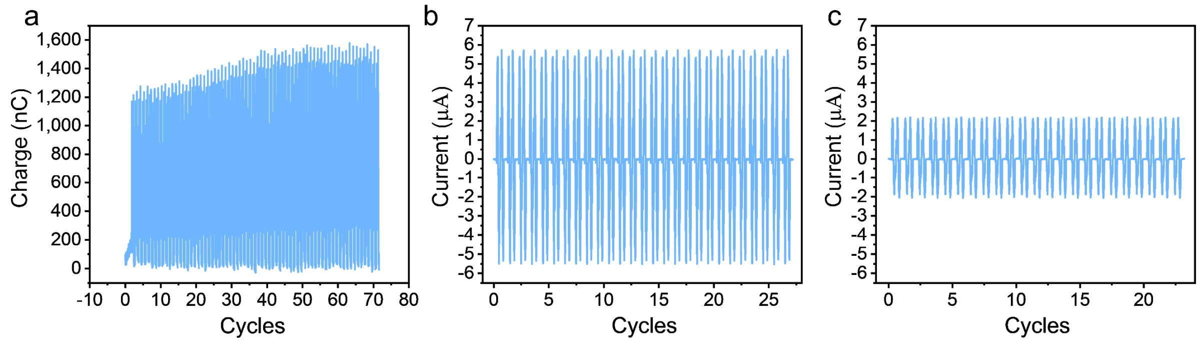

Figure S14. The output waveforms of S-TENG in air at different stage in the cycles. (a) The output charge of S-TENG in the first 70 cycles. The short-circuit of S-TENG after (b) 50 cycles and (c) 40k cycles.

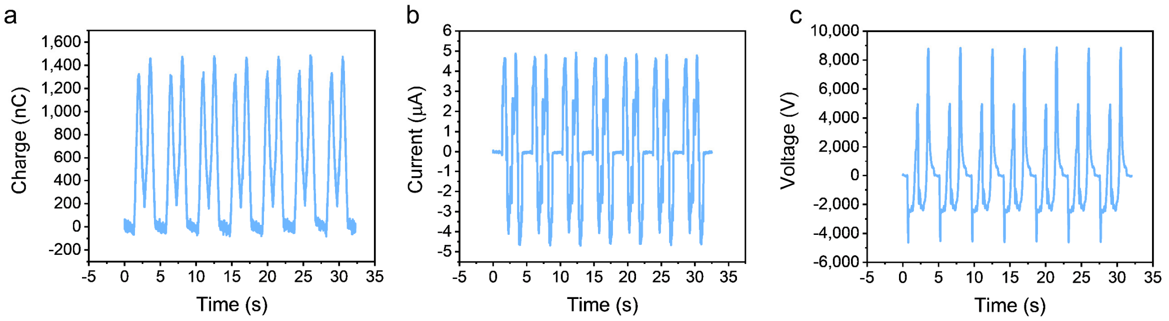

Figure S15. The output performance of S-TENG in air. (a) The output charge, (b) short-circuit current and (c) voltage of S-TENG at sliding speed of 0.05 m s^{-1}.

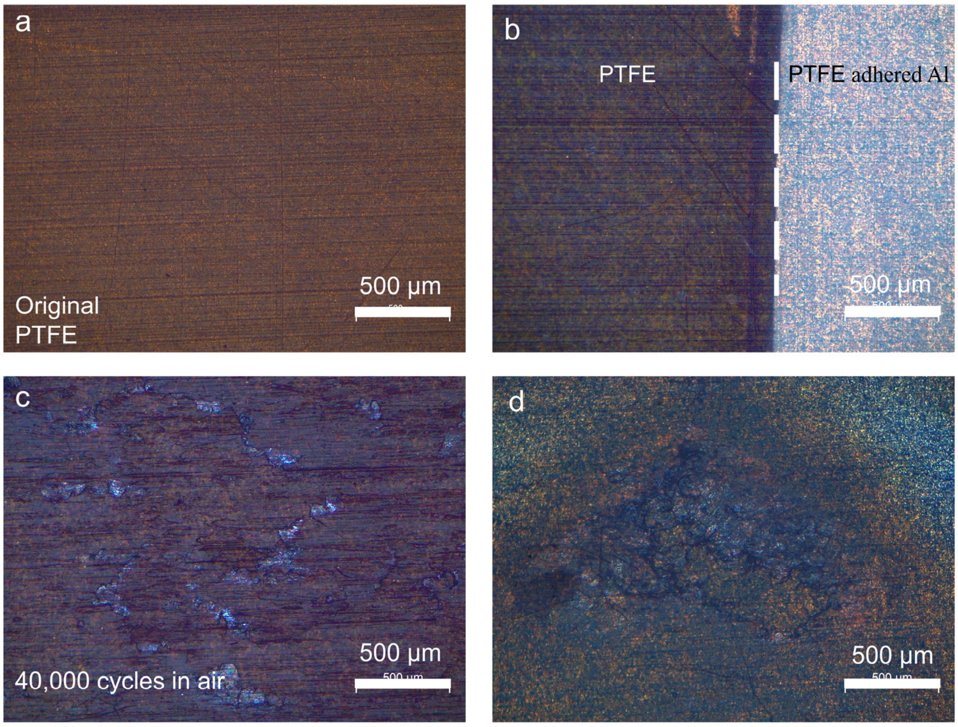

Figure S16. Characterization of the surface wear for PTFE. (a) The surface microscopic image of original PTFE. (b) The surface microscopic image of original PTFE adhered to electrode. (c) The concave scratches and (d) distributed debris on the surface of PTFE after 40k cycles.

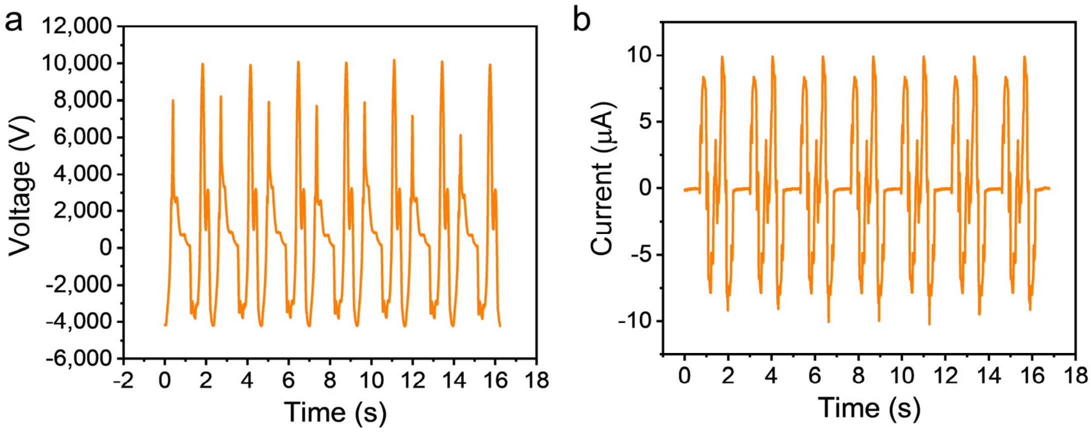

Figure S17. The output performance of LP-TENG. (a) The voltage and (b) short-circuit current of LP-TENG at sliding speed of 0.1 m s^{-1}.

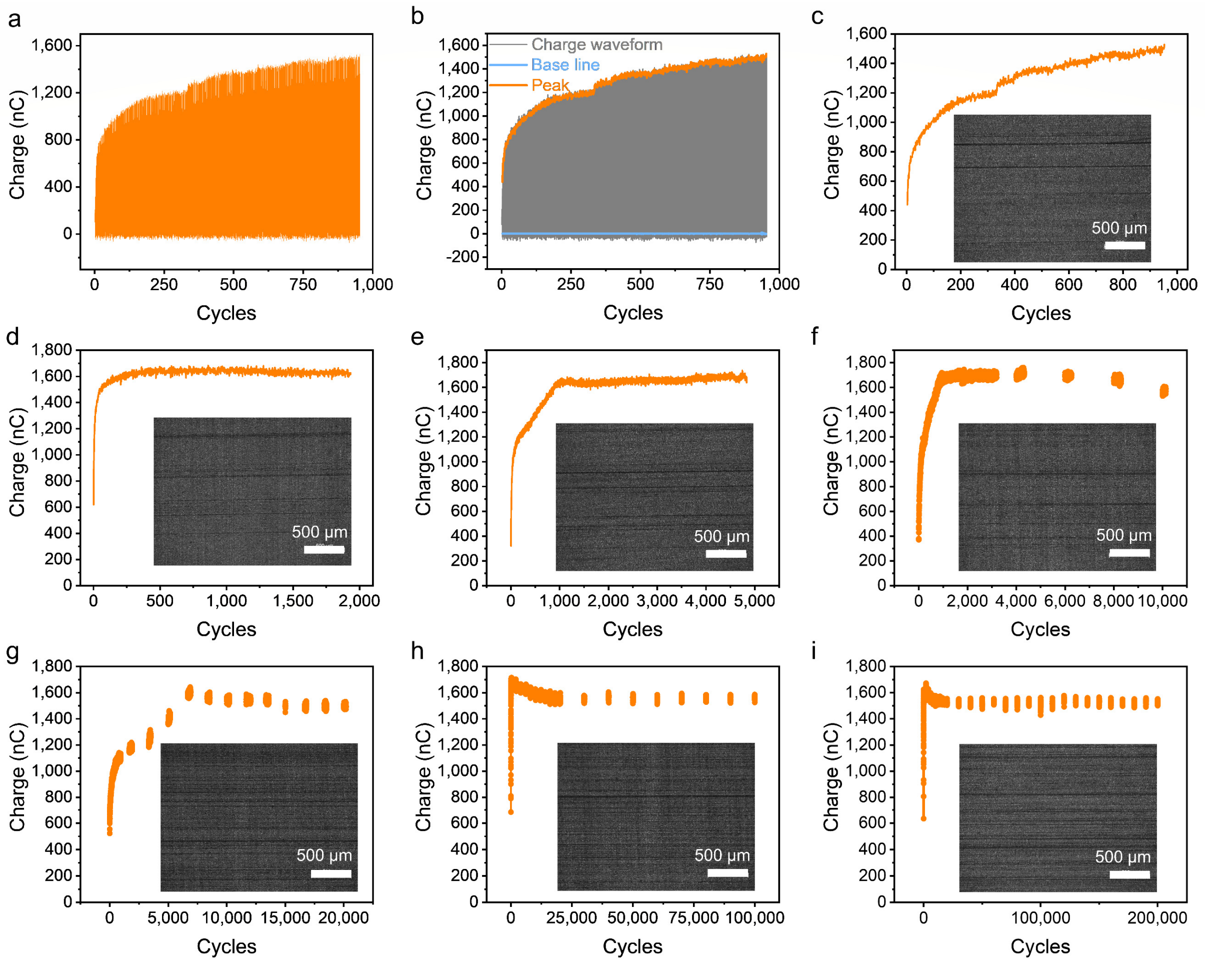

Figure S18. The long-term durability of LP-TENG. (a) The original output charge waveform of 950 operation cycles. (b) The peaks and base line of the original waveform. (c) The curve difference between peaks and base line. a-c) Showing the method of data processing. (d-i) The output charge of LP-TENG within different operation cycles (2,000, 5,000, 10,000, 20,000, 100,000, 200,000 cycles). Insets are the surface microscopic images of the nylon after cycles.

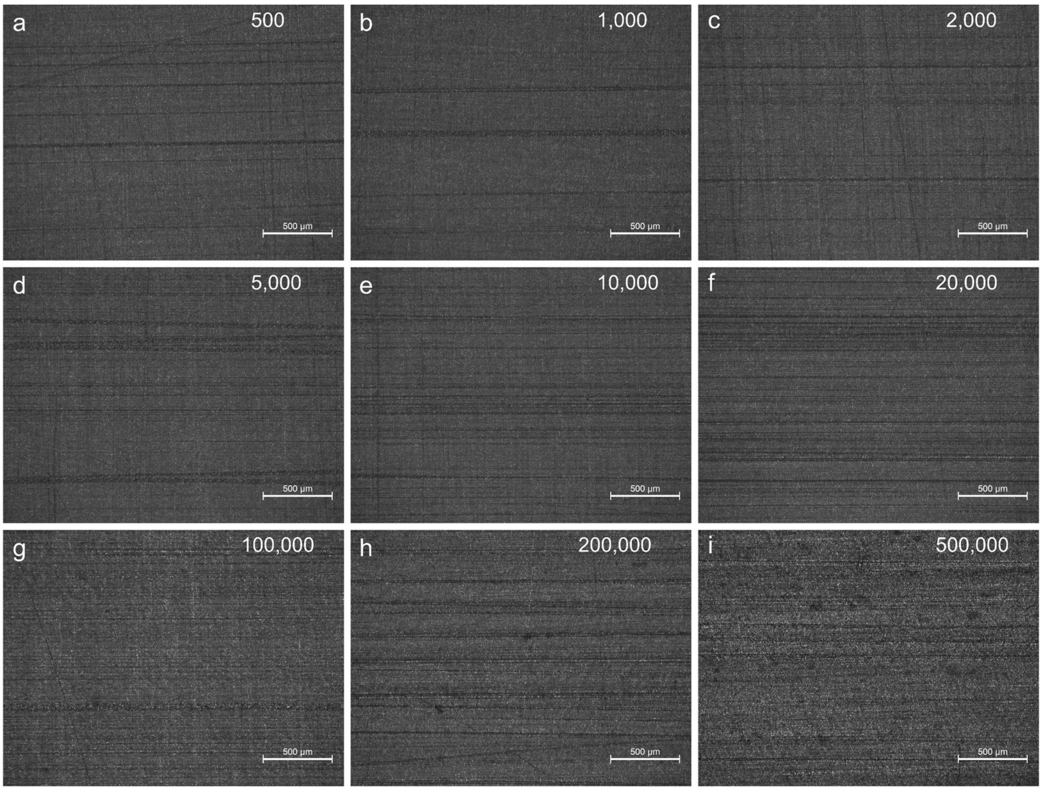

Figure S19. The surface microscopic images of PTFE after different operation cycles. The wear marks on the surface gradually tend to be constant after about 20k cycles.

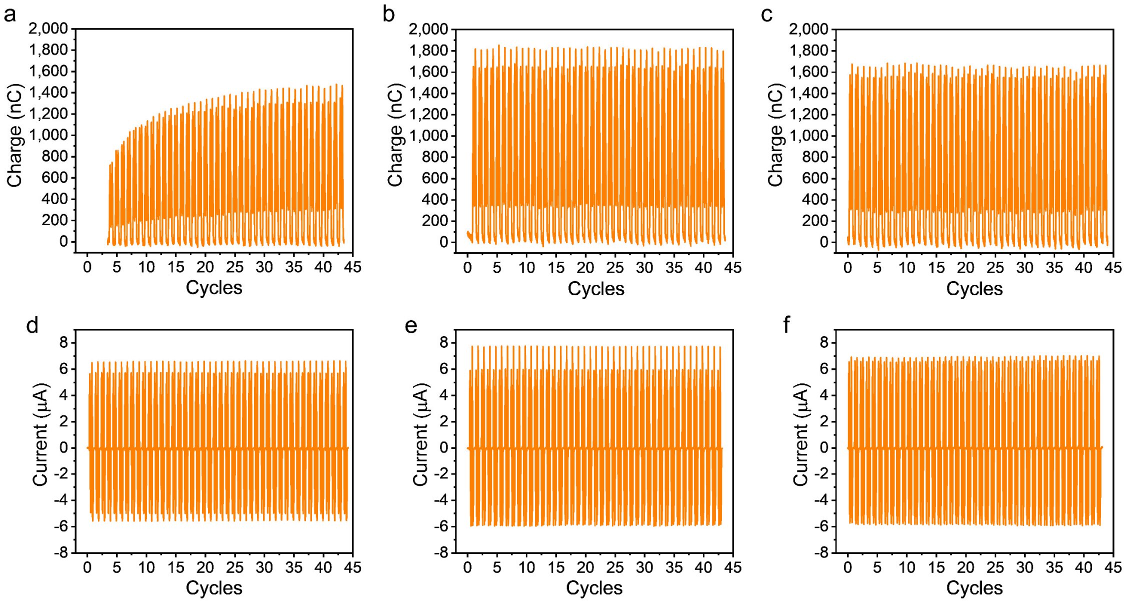

Figure S20. The output waveforms of LP-TENG at different stage in the 500,000 cycles. (a-c) The output charges and (d-f) short-circuit currents of LP-TENG at the first 40 cycles, the maximum output stage and last 40 cycles, respectively.

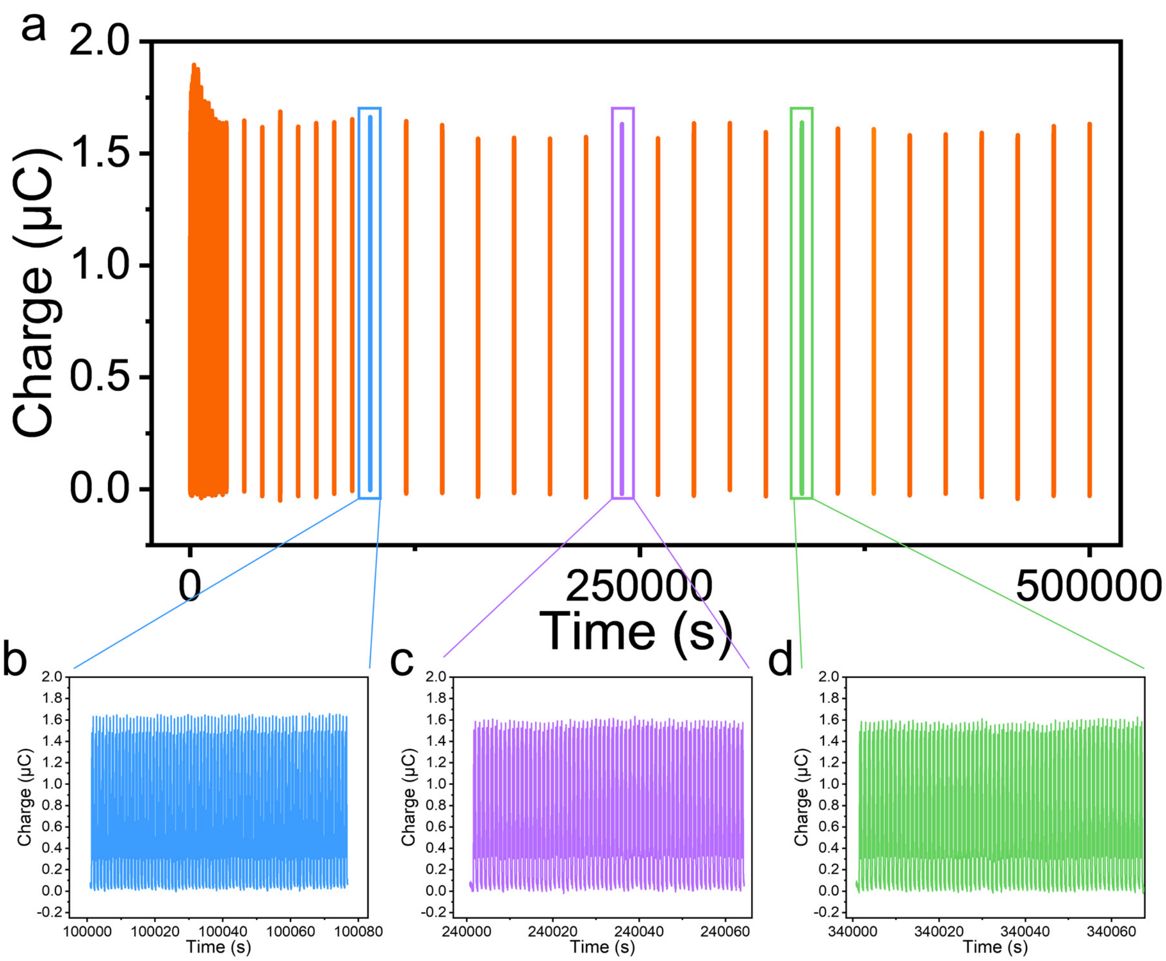

Figure S21. The detail waveform of 500,000 cycles durability test. (a) The original output charge waveform in 500,000 cycles durability test. (b-d) The enlarged output charge waveforms in (a).

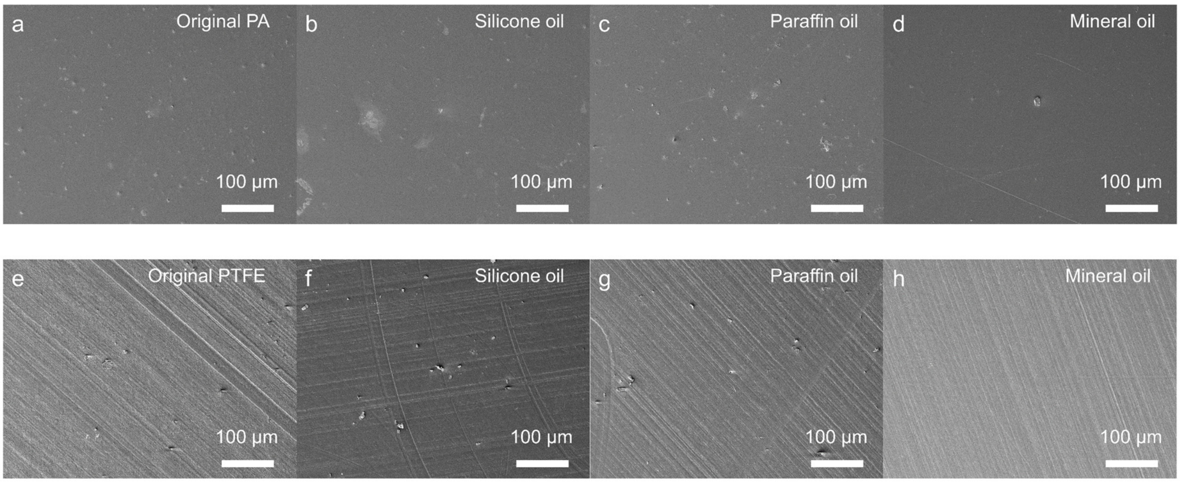

Figure S22. The SEM images of PTFE and nylon treaded by different liquid. The SEM images of (a-d) PA and (e-h) PTFE treaded by air (the original one), silicone oil, paraffin oil and mineral oil, respectively.

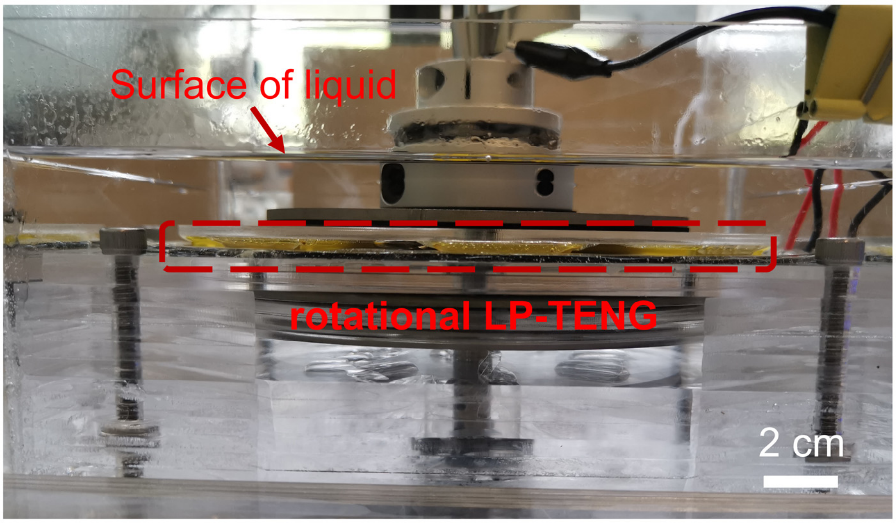

Figure S23. Photograph of rotational LP-TENG.

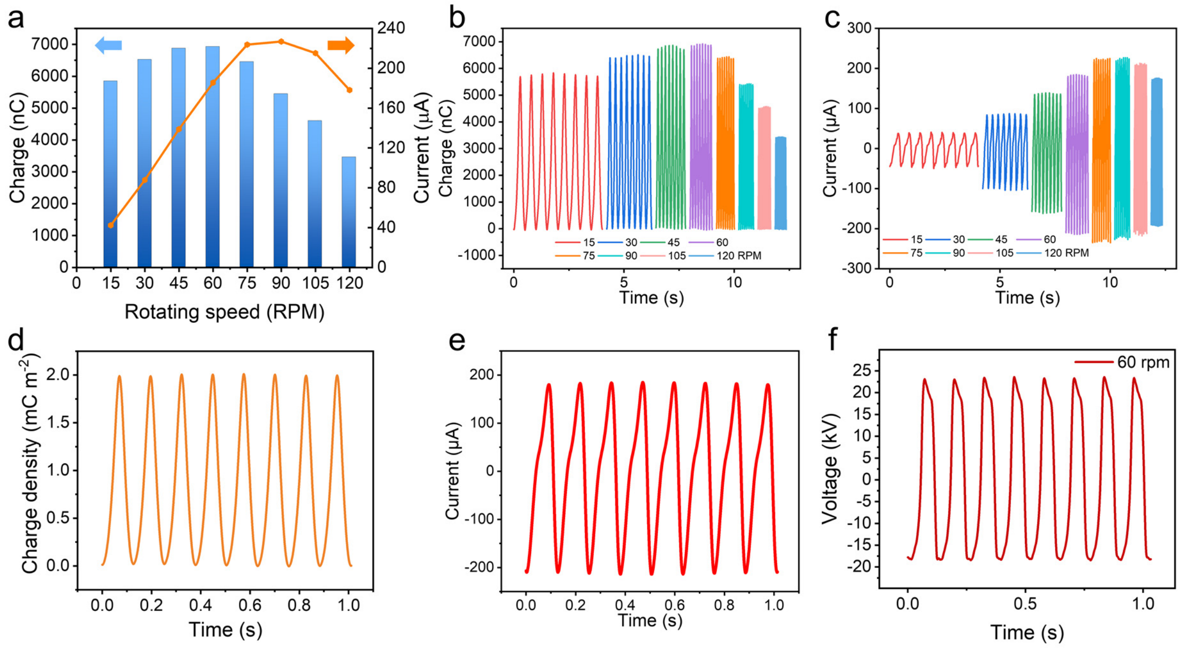

Figure S24. The output performance of rotary LP-TENG. (a) The output charge and current of rotary LP-TENG. (b-c) The detail waveform of output charge and current at different rotating speed (15 to 120 rpm). (d) The charge density, (e) short circuit current and (f) voltage of rotary LP-TENG at 60 rpm.

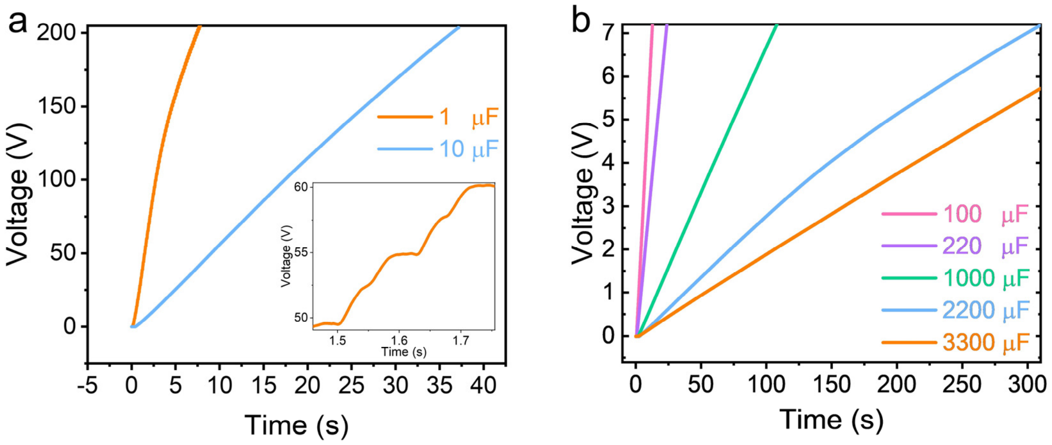

Figure S25. The voltage curves of charging different capacitors. The inset in (a) is the enlarged view of the charging voltage step of 1 μF capacitor.

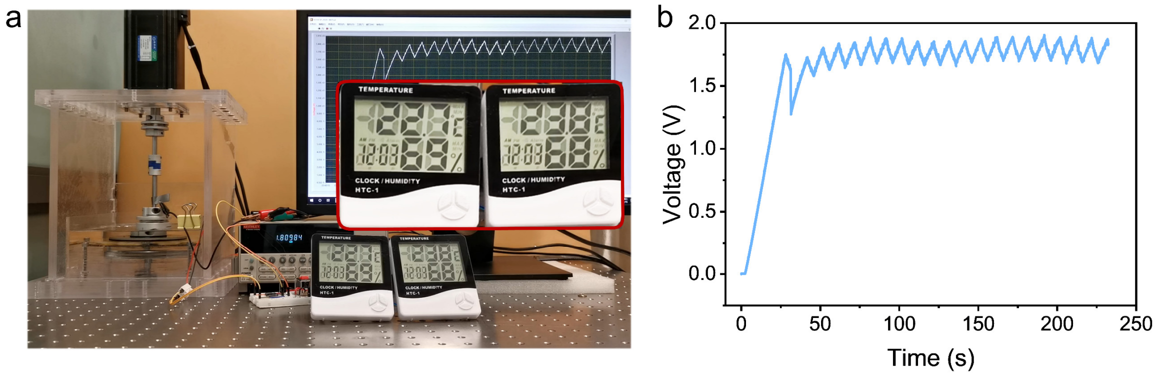

Figure S26. Powering two commercial hygro-thermometers. (a) Charging 1 mF capacitor while powering two commercial hygro-thermometers in parallel at low rotating speed of 45 rpm. (b) The charging curve of powering hygro-thermometers.

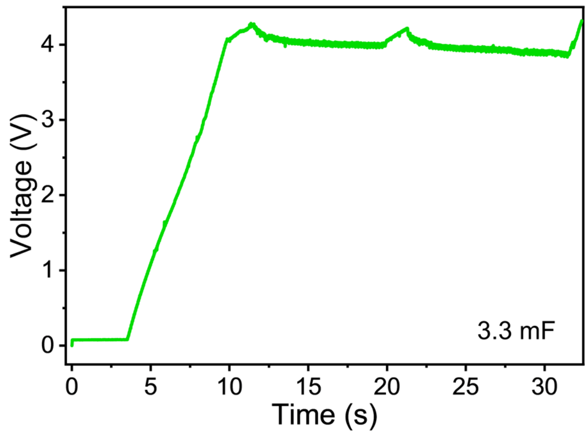

Figure S27. The charging curve of a 3.3 mF capacitor during the cell phone charging process.

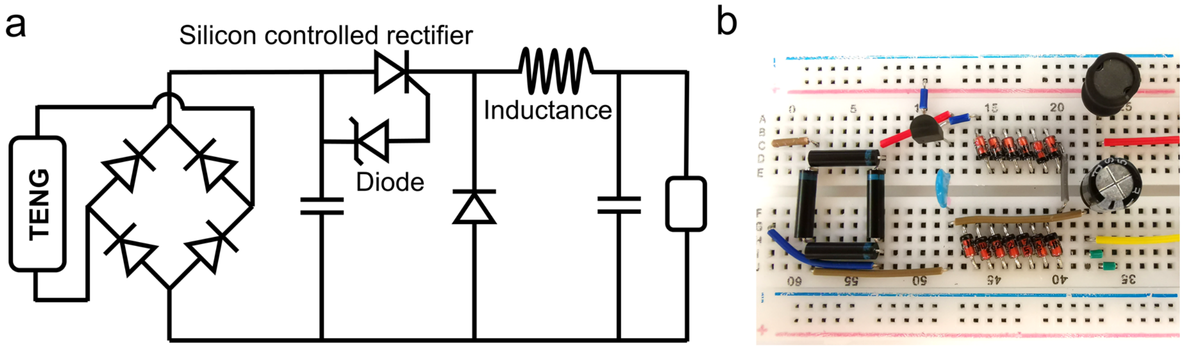

Figure S28. The circuit diagram and photo of power management circuit.

Supporting Tables

Table S1. The parameters of lubrication liquids.

Name	Water content (μL/L)	Breakdown voltage (kV)	Permittivity
Silicone oil	41.3	48	2.418
Mineral oil	15.7	50.2	2.070
Transformer oil	15.3	55.6	2.015
Paraffin oil	14.1	41	2.045

Table S2. Parameters of silicone oil with different viscosity.

Kinematic viscosity (cst)	Dielectric loss factor (%)	Permittivity	DC resistivity (Ω·m)
0.65 (363 K)	0.008	2.438	2.06×10^{13}
0.65 (333 K)	0.015	2.527	1.28×10^{13}
5 (333 K)	0.013	2.539	2.55×10^{13}
10 (333 K)	0.002	2.525	4.15×10^{13}
20 (333 K)	0.004	2.517	1.81×10^{13}
30 (363 K)	0.007	2.454	7.36×10^{12}
30 (333 K)	0.006	2.535	1.131×10^{13}
40 (333 K)	0.001	2.565	1.04×10^{13}
50 (333 K)	0.001	2.597	4.81×10^{13}
100 (363 K)	0.006	2.517	1.78×10^{13}
100 (333 K)	0.001	2.610	5.51×10^{13}

Table S3. Output performance comparison with the reported S-TENG.

Name	Mode	Charge density ($mC\ m^{-2}$)	Power density ($W\ m^{-2}\ Hz^{-1}$)	Frequency (Hz)	Units	References
LLS-TENG	Rotary	NA	22.59	1.66	6	Ref.40 (liquid lubrication)
HS-TENG	Sliding	0.026	NA	0.625	3	Ref.37 (liquid lubrication)
TENG	Sliding	0.15	NA	1	1	Ref.39 (liquid lubrication)
FS-TENG	Sliding	0.4	NA	0.55	1	Ref.38 (liquid lubrication)
	Rotary	0.094	3.45	1.5	48	
MA-S TENG	Sliding	0.092	4.5	1	15	Ref.20
TEG	Rotary	0.04	3.8*	50	60	Ref.41
FTENG	Sliding	0.065	8.375	0.8	1	Ref.32
RC-TENR	Rotary	0.14	1.242*	2	48	Ref.26
CPS-TENG	Sliding	1.328	0.265	2.5	1	Ref.27
CSA-TENG	Sliding	1.63	1.3	1	1	Ref.29
	Rotary	0.71	8	1	12	
LP-TENG	Sliding	1.96	4.43	0.45	1	This work
	Rotary	2.0	87.26*	1	8	

* Average power density

Supporting Notes

Note S1. The mechanism of charge space accumulation (CSA) strategy.

Figure S1(a) shows the basic structure of CSA-TENG. Different from traditional free-standing mode TENG, it has a grounded top electrode on the back of the sliding layer (PTFE) and two extended area (PA) without bottom electrodes. When the top electrode is not connected to the ground and the sliding range is restrained as depicted in Figure S1(b), it is the case of traditional free-standing mode TENG. The mechanism of CSA includes two parts, one is the grounded top electrode can suppress the inner electric field between PTFE and PA layer, the other is to use the charge dissipation feature of PA layer. Typically, the grounded top electrode can decrease the inner electric field between PTFE and PA layer, so that to avoid triboelectric charge recombination through air breakdown. In this way, the surface charge density on PTFE layer can be increased (Figure S1(c)). It is worth noting that the bottom electrode can grasp the charges on the overlapped PA surface and prevent them from dissipating. So, when the charge density on PA layer is saturated, PTFE will not gain more charges for energy conversion. In this case, the extended PA area without the bottom electrode is designed. When PTFE sliding on the extended area, extra negative charges are accumulated on PTFE surface (Figure S1(d)). When it slides out from the extended area, positive charges on PA surface dissipate, so that the surface can offer charges again during the next triboelectrification process (Figure S1(e-f)). Based on the process described above, large amount of charges can be accumulated on PTFE surface and improve the output performance of TENG. The detailed description, some theoretical and experimental proof can be found in our previous work [29].

Note S2. Effect of materials, oil viscosity and sliding speed on the output of LP-TENG.

Silicone oil is the best candidate for high performance LP-TENG in our experiments (Figure S4). Besides the categories of insulating liquid medium, tribo-materials, liquid viscosity and operating speed also affect the output performance of LP-TENG. In the experiment, we measured PTFE/PA, FEP/PA and Kapton/PA tribo-pairs. Here, PA was always used as the tribo-material for the stationary part due to its charge dissipation property for CSA mechanism. As shown in Figure S7(a) and S7(b), PTFE/PA had the highest output, which was adopted in our experiments.

The kinematic viscosity of the lubricating liquid is also critical to the output performance. The kinematic viscosity of liquid is related to its ability of flow. The greater the kinematic viscosity, the longer the response time of the flow, and the worse the fluidity. The charge transmission in the liquid depends on the good fluidity, as a consequence, the liquid with bigger kinematic viscosity is not conducive to the charge transmission, thus limiting the output performance of LP-TENG as shown in Figure S7(c). For oil, its kinemical viscosity is related to its molecular weight, the larger the molecular weight, the greater the kinemical viscosity. The extended blank triboarea, which plays a key role in triboelectrification for LP-TENG, needs to dissipate the surface charge to the outside, and the liquid with small molecular weight is more conducive to this behavior, so the liquid with low kinetic viscosity is more beneficial to the output performance of LP-TENG.

In addition, as presented in Figure 2(c), the gap distance (thickness of the insulating oil between two solid interface) affects both the charge-liquid transmission process and the intrinsic output of TENG device. In this case, at a constant sliding speed, a thicker liquid layer is formed with a higher viscosity of silicone oil, which would lower the electric output of LP-TENG (Figure S7(c)). Accordingly, with a certain silicone oil, a thicker liquid layer is formed at a higher sliding speed, which would also lower the electric output of LP-TENG (Figure S7(d) and (e)). Therefore, a silicone oil with small viscosity (0.65 cst) is employed in our experiments, and lower working speed for higher efficiency also matches the goal of low frequency energy harvesting.

Note S3. Simulation the effect of VB bar.

In order to study the effect of VB bar on the open-circuit output voltage of LP-TENG theoretically, the potential distribution simulation based on finite element analysis (COMSOL Multiphysics) was carried out for demonstrating. Figure S8(a) and (b) depict the physical model and the related parameters for simulation respectively. In which, D represents the distance between the edge of bottom electrode and VB bar. And the sliding distance is defined as the distance from the left end of PA layer to the left end of PTFE layer. Before simulating, zero-voltage state is set at where the sliding part locating on left bottom electrode exactly (sliding distance: 14 mm). Later, the simulation is proceeded by scanning the sliding distance from 0 to 40 with 1 mm step. Figure S8(c) presents the simulated potential distribution of LP-TENG with D varying from 0 to 10 mm when sliding part at the distance of 26 mm. From which, we can see that, with the utilization of VB bar, the intensive electric potential around bottom electrodes is dispersed. And the dispersing effect is enhanced when the D of VB bar increasing. In addition, the potential difference between two bottom electrodes at each sliding distance with various D of VB bar are shown in Figure S8(d), the open-circuit voltage of LP-TENG could be suppressed by VB bar, which match well with the experimental results.

Note S4. Evaluating the energy conversion efficiency and crest factor.

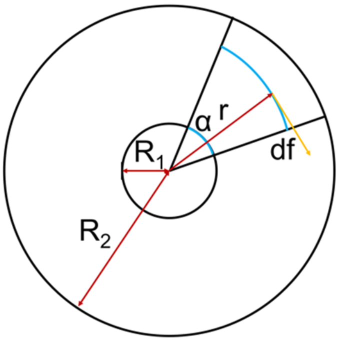

For sliding or rotating mode TENG, the output electricity comes from working done against friction. The total external input energy should include the work done against friction, the output electrical energy and rotational kinetic energy. To know the energy which used to against the friction, we should calculate the friction moment M, as follow.

$$M = \int \boldsymbol{r} \times d\boldsymbol{f} \quad \text{(S1)}$$

Where $\boldsymbol{r}$ is the radius of rotor, $d\boldsymbol{f}$ is the elementary friction.

Due to the radius $\boldsymbol{r}$ is perpendicular to the elementary friction d$\boldsymbol{f}$, so friction moment M can be written as follow.

$$M = \int r df \quad \text{(S2)}$$

The elementary friction can be described as follow.

$$df = \mu dF_N \quad \text{(S3)}$$

Where μ is the dynamic friction factor, F_N is the positive pressure, can be described as follow.

$$F_N = \frac{1}{2} p\alpha a\left(R_2{}^2 - R_1{}^2\right) \quad \text{(S4)}$$

Where p is the number of sectors on the rotor, α is the center angle of the sector, a is the pressure, R_1 and R_2 are the inner and outer radius of the rotor respectively.

The inner radius R_1 is a definite constant and the r is between R_1 and R_2. Differential the positive pressure F_N to obtain the elementary positive pressure dF_N as follow.

$$dF_N = ap\alpha r dr \quad \text{(S5)}$$

Substituting Supporting Equation (S3) and Supporting Equation (S5) into Supporting Equation (S2), the friction moment M can be written as follow.

$$M = \int_{R_1}^{R_2} ap\alpha\mu r^2 dr \quad \text{(S6)}$$

Finish the integrate of Supporting Equation (S6), the friction moment M described as follow.

$$M = \frac{2}{3} \mu F_N \frac{R_2{}^3 - R_1{}^3}{R_2{}^2 - R_1{}^2} \quad \text{(S7)}$$

The relationship of the energy used to against the friction in one rotation E_f and friction moment M as follow.

$$E_f = \int_0^{2\pi} Md\theta \tag{S8}$$

Where the θ is the rotation angle.

Substituting Supporting Equation (S7) into Supporting Equation (S8) and integrate, the E_f is obtained as follow.

$$E_f = \frac{4}{3}\pi\mu F_N \frac{{R_2}^3 - {R_1}^3}{{R_2}^2 - {R_1}^2} \tag{S9}$$

The rotational kinetic energy E_k can be described as follow

$$E_k = \frac{1}{2} I\omega^2 \tag{R10}$$

Where the ω is the angular velocity and I is the rotational inertia, which can be described as follow

$$I = \frac{1}{2} m\left({R_1}^2 + {R_2}^2\right) \tag{R11}$$

Where the m is mass of rotor, R_1 and R_2 are the the inner and outer radiuss of the rotor respectively.

As a result, the rotational kinetic energy E_k can be described as follow

$$E_k = \frac{1}{4} m\left({R_1}^2 + {R_2}^2\right)\omega^2 \tag{R12}$$

Hence, the energy conversion efficiency η can be calculated as follow.

$$\eta = \frac{W_e}{E_f + W_e + E_k} \tag{S13}$$

Where W_e is the output electricity in one rotation, described as follow.

$$W_e = P_{av} \cdot t \tag{S14}$$

Where P_{av} is the average output power and the t is time.

Hence, the energy conversion efficiency η can be described as follow.

$$\eta = \frac{W_e}{E_f + W_e + E_k} = \frac{P_{av} \cdot t}{\frac{4}{3}\pi\mu F_N \frac{{R_2}^3 - {R_1}^3}{{R_2}^2 - {R_1}^2} + P_{av} \cdot t + \frac{1}{4} m\left({R_1}^2 + {R_2}^2\right)\omega^2} \tag{S15}$$

For the rotating mode TENG which working at 60 rpm, W_e is 302 mJ, μ is 0.04324, F_N is 20.65 N, m is107g, ω is 2π s^{-1}, R_1 and R_2 are 6 mm and 83 mm respectively. Hence, the energy conversion efficiency η is up to 48.61%.

The current crest factor K_p can be calculated as follow

$$K_p = \frac{I_{peak}}{I_{rms}} \tag{S16}$$

Where I_{peak} and I_{rms} are the peak value of current and the effective value of current, respectively. I_{rms} can be described as follow

$$I_{rms} = \sqrt{\frac{1}{t_2 - t_1} \int_{t_1}^{t_2} I(t)^2 dt} \tag{S17}$$

Where t_1 and t_2 are the start and end times of one working cycle.

Hence, the current crest factor K_p can be described as follow

$$K_p = \frac{I_{peak}}{\sqrt{\frac{1}{t_2-t_1}\int_{t_1}^{t_2} I(t)^2 dt}} \tag{S18}$$

Taking the current of rotatory LP-TENG at 60 RPM in Figure S24(e) as an example, t_1 is 0.419 s, t_2 is 0.546 s, I_{rms} is 0.129 mA and I_{peak} is 0.185 mA. Its creat factor is calculated as 1.43 according to the Supporting Equation (S18).
